# Adaptive robot mediated upper limb training using electromyogram-based muscle fatigue indicators

**DOI:** 10.1371/journal.pone.0233545

**Published:** 2020-05-29

**Authors:** Azeemsha Thacham Poyil, Volker Steuber, Farshid Amirabdollahian

**Affiliations:** School of Computer Science, University of Hertfordshire, Hatfield, United Kingdom; University of Bourgogne France Comté, FRANCE

## Abstract

Studies on improving the adaptability of upper limb rehabilitation training do not often consider the implications of muscle fatigue sufficiently. In this study, electromyogram features were used as fatigue indicators in the context of human-robot interaction. They were utilised for auto-adaptation of the task difficulty, which resulted in a prolonged training interaction. The electromyogram data was collected from three gross-muscles of the upper limb in 30 healthy participants. The experiment followed a protocol for increasing the muscle strength by progressive strength training, that was an implementation of a known method in sports science for muscle training, in a new domain of robotic adaptation in muscle training. The study also compared how the participants in three experimental conditions perceived the change in task difficulty levels. One task benefitted from robotic adaptation (Intervention group) where the robot adjusted the task difficulty. The other two tasks were control groups 1 and 2. There was no difficulty adjustment at all in Control 1 group and the difficulty was adjusted manually in Control 2 group. The results indicated that the participants could perform a prolonged progressive strength training exercise with more repetitions with the help of a fatigue-based robotic adaptation, compared to the training interactions, which were based on manual/no adaptation. This study showed that it is possible to alter the level of the challenge using fatigue indicators, and thus, increase the interaction time. The results of the study are expected to be extended to stroke patients in the future by utilising the potential for adapting the training difficulty according to the patient’s muscular state, and also to have a large number repetitions in a robot-assisted training environment.

## Introduction

Human-robot interaction (HRI) is the process of humans and robots working together to accomplish a goal to make the interaction beneficial to humans. While designing an HRI scheme, it is essential to understand the users of the system and evaluate the capabilities of humans and robots. An acceptable HRI solution is expected to be adaptable by detecting and responding to the changes in the environment and its users [1]. Hence, an adaptive robotic interaction will require better sensing of the human performance parameters [2].

Stroke patients during physical therapies may easily come to a state of fatigue due to their reduced muscular and cognitive capabilities. Rehabilitation robotics can be used for training stroke survivors to improve their muscular disabilities through regular exercises. Robots have a potential to be used in strength training and rehabilitation [3, 4, 5, 6]. Robots are widely adopted in strength training since they can increase the efficiency of the interaction by collecting performance data, and thus, assess the progress of the training sessions. They have an excellent potential to assist rehabilitation therapists in providing training for extended periods and can help to deliver a large number of repetitions in training exercises. Robots enable multiple patients to be treated at the same time and possibly even remotely [3].

Increased exercise duration has been reported to have helped stroke recovery [7]. Increasing the number of task repetitions is also known to help motor relearning significantly, and thus, help the recovery of upper limb functions [8, 9, 10, 11]. Repetitions in training exercises are thought to impact on their neuro-plasticity [12, 13, 14], but the repetitions often result in a faster occurrence of muscle fatigue. Acute adaptations (like fatigue) often affect adversely during rehabilitation training if not considered sufficiently. If a stoke patient feels a state of muscle fatigue, the training session may have to be paused to allow recovery or abandoned due to the restrictions put by ethics committee limiting the maximum time for a rehabilitation session. The lost training session might also be demotivating to the patient [15]. Existing physical therapies are designed without sufficiently considering the implications of fatigue to the patient. So, researchers in the field of robot-assisted rehabilitation have been trying to address the adaptability of rehabilitation training according to the physical state of the patients. Given this, identifying if a better outcome can be achieved during a rehabilitation training by adapting to individual muscular status, i.e. with respect to fatigue, is a novel area of research.

Progressive strength training is an exercise that builds physical strength, especially in weak or injured body parts, through a progressively difficult task according to a formula based on the subject’s maximum strength at the starting point [16]. Progressive muscle training exercises can help to have chronic adaptations (like maximal voluntary contraction) over time. Chronic adaptations are the long-term physiological changes that take place in the human body as a result of participating in a training program. The types of adaptations that lead to improved performance are dependent on the specific type of training that is undertaken. The techniques of progressive resistance exercise suggest to perform many repetitions until fatigue, then allow sufficient rest between exercises for recovery, and then, to increase the resistance as the ability to generate force increases [17, 18]. Robot-assisted strength training can be effective if it is progressive and challenging based on the person’s abilities [19, 20].

Rehabilitation training for stroke patients is suggested to involve protocols for developing muscle strength and relearning the lost motor skills. A rehabilitative training is considered to be useful if it can help the patients to train more at the initial stages of recovery and to make the task progressively challenging. Progressive resistance training can be the most effective treatment to improve the muscle strength in patients, and studies suggest that there are long-term benefits for this [21, 22, 23]. Resistance training/functional muscle strengthening is found to be helpful for rehabilitation, and there is evidence to support “increased dose” of exercises for stroke rehabilitation. Many studies [24, 25, 26, 7] have suggested that stroke rehabilitation can benefit from intensive muscle training, and a progressive strength training is helpful for stroke recovery by improving the muscle strength. However, there is no universally accepted protocol available for the upper limb rehabilitation of stroke patients, and the treatment programs vary in the duration, intensity, and frequency of the rehabilitative therapy [25]. While this research is interested in identifying interaction fatigue, there were no accepted procedures from the stroke therapy literature that could be used towards inducing fatigue. Hence we relied on literature from sports science [27], where both progressive strength training and fatigue have been featured.

Previous work by Octavia et al. [28] has explored the muscle fatigue developed in the participants during a robotic training interaction, and the proposed EMG based features indicated muscle fatigue significantly in all the participants. However, the fatigue indicators were not used for any robotic adaptation. In a different study, a strategy for adapting the physical behaviour of a robot according to the muscle fatigue in human-robot co-manipulation tasks was suggested by Paternel et al. [29]. In the study, the robot initially imitated the human to perform a collaborative task in a leader-follower manner, using feedback about human motor behaviour. The robot also simultaneously learned the skill in an on-line fashion. When a fatigue threshold was reached, the robot used the learned skill to take over the task, which reduced the human effort. However, the study was not designed for a context of rehabilitation. Besides the “help-if” adaptive modality used here, other control strategies such as challenging, challenging-then supporting, supporting, under-supporting and under-challenging have also been explored [30]. In our previous study, we have assessed muscle fatigue in healthy subjects during an assist-as-needed interaction with the HapticMaster robot using Electromyogram (EMG) features [31]. The EMG features, average power and median power frequency (MPF) were used as fatigue indicators. The average power of the EMG signal is defined as the energy contained in the EMG signal over a specified time interval [32]. Median frequency or MPF is defined as the frequency, which separates the power spectrum of signals in two equal regions with the same power [33, 34]. Past studies have explored both mean power frequency (MNF) and median frequency to detect muscle fatigue [35, 36, 37, 38, 39, 40]. It is reported that MNF is slightly higher than the median frequency because of the skewed shape of the EMG power spectrum. In contrast, the variance of MNF is typically lower than that of median frequency [34]. However, the estimation of median frequency is less affected by random noise, particularly in the case of noise located in the high-frequency band of the EMG power spectrum [34]. In the initial study, during muscle fatigue, statistically significant trends in the average power and median frequency were observed in the involved muscles. These provided the material for the current study, where the fatigue indicator (median frequency) was used to change the interaction difficultly during human-robot interaction automatically.

So, the idea here is to combine the above concepts for a potential application in stroke rehabilitation. The procedure for inducing muscle fatigue can be adopted from sports science literature for muscle training [27]. The task difficulty levels can be quantified based on the maximum voluntary strength of participants and use a proportional increment of the difficulty at regular intervals [41]. While the volume of repetitions is important for motor learning, the progressive resistance used to challenge a subject is key for strengthening. Moreover, robotic stroke rehabilitation studies such as [42] and [30] have suggested strategies of reducing task difficulty, when high fatigue is detected. A balance between supporting and challenging (“challenging-then-supporting” sessions) has been suggested by Basteris et al. [43] to allow optimal learning. Hence, a prolonged progressive exercise (with a high volume of repetitions), which reduces difficulty during the onset of fatigue, and then, progressively increases the difficulty when “not-fatigued”, is proposed here for muscle strength training in stroke patients. The idea is to prevent the stroke patients from going to a high state of fatigue, that would otherwise make them demotivated from continuing the training, or may cause further damage to their muscles. By providing motivational elements (haptic feedback, visuals, etc.) in addition to the adapting challenge levels, the protocols can be made to have better potential for applications in rehabilitation training. Hence, the study was designed to explore the robot-assisted incremental strength training, which would adapt the task difficulty based on the run-time muscular state of the participants through fatigue indicators. The EMG based fatigue indicators were used to enable the fatigue-adaptive training interaction allowing a prolonged exercise cycle. The participants were expected to perform a prolonged progressive strength training exercise with more repetitions with the help of a fatigue-based robotic adaptation compared to the training interactions, which were based on manual/no adaptation.

## Materials and methods

**Fig 1:**
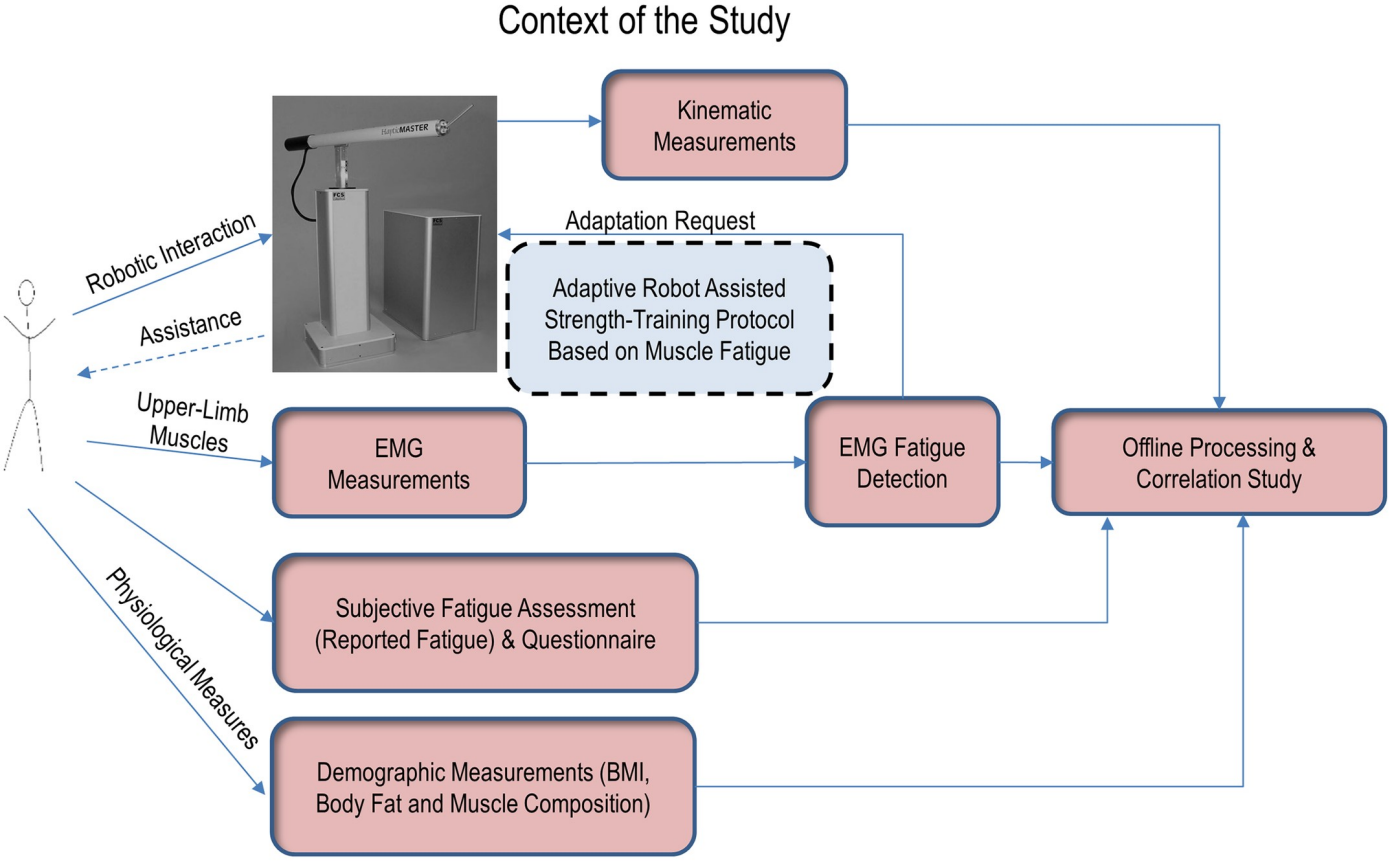
Context of the study. HapticMaster robot was configured to adapt its environment based on the detected muscle fatigue. EMG measured from upper limb muscles was used to detect the fatigue. The adaptive strength training algorithm forms a closed loop, as shown. Kinematic measurements, detected fatigue, reported fatigue, and demographic measurements were also analysed off-line.

The overall context of the experiment is explained in Fig 1.

### Experimental setup

**Fig 2:**
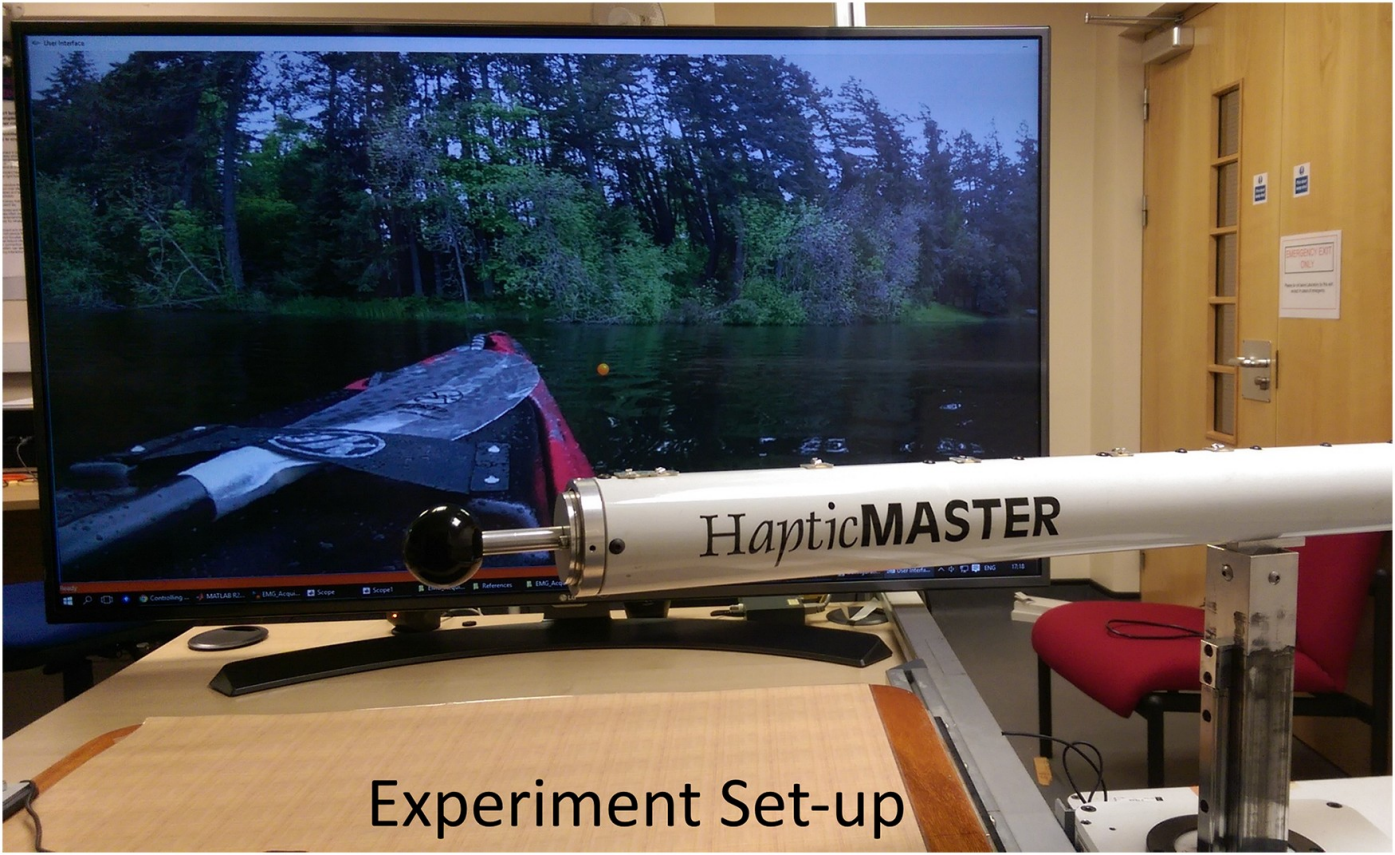
Experimental setup. The experimental setup included HapticMaster robot, visual guidance and animated background. The front-end of the rowing boat was shown on the LCD display in front of the participant. The rowing environment was embedded with audio cues and haptic sensation of underwater viscosity.

The experimental setup included the HapticMaster robotic interface configured for a rowing task. HapticMaster robot was configured to adapt its environment based on the fatigue detected from the upper limb muscles of the participants. The EMG measured from the muscles was used to detect the fatigue. An animated rowing environment embedded with audio cues and haptic sensation of underwater viscosity were created using the HapticMaster robot, to support an aesthetically pleasing interactive task for the participants. A user-interface and the animated environment were developed using Visual C++ and with Open GL programming in a Windows PC. The background on a wide-screen 43 inch LCD monitor would display the front-end of a rowing boat with flowing water as in Fig 2, which would potentially motivate the participants for active involvement in the task. Suitable audio for water flow was played in the background. The HapticMaster robot was programmed to deliver different viscosities underwater and above water while rowing. The starting time, the break period and the stopping time of the experiment were guided by audio cues.

**Fig 3:**
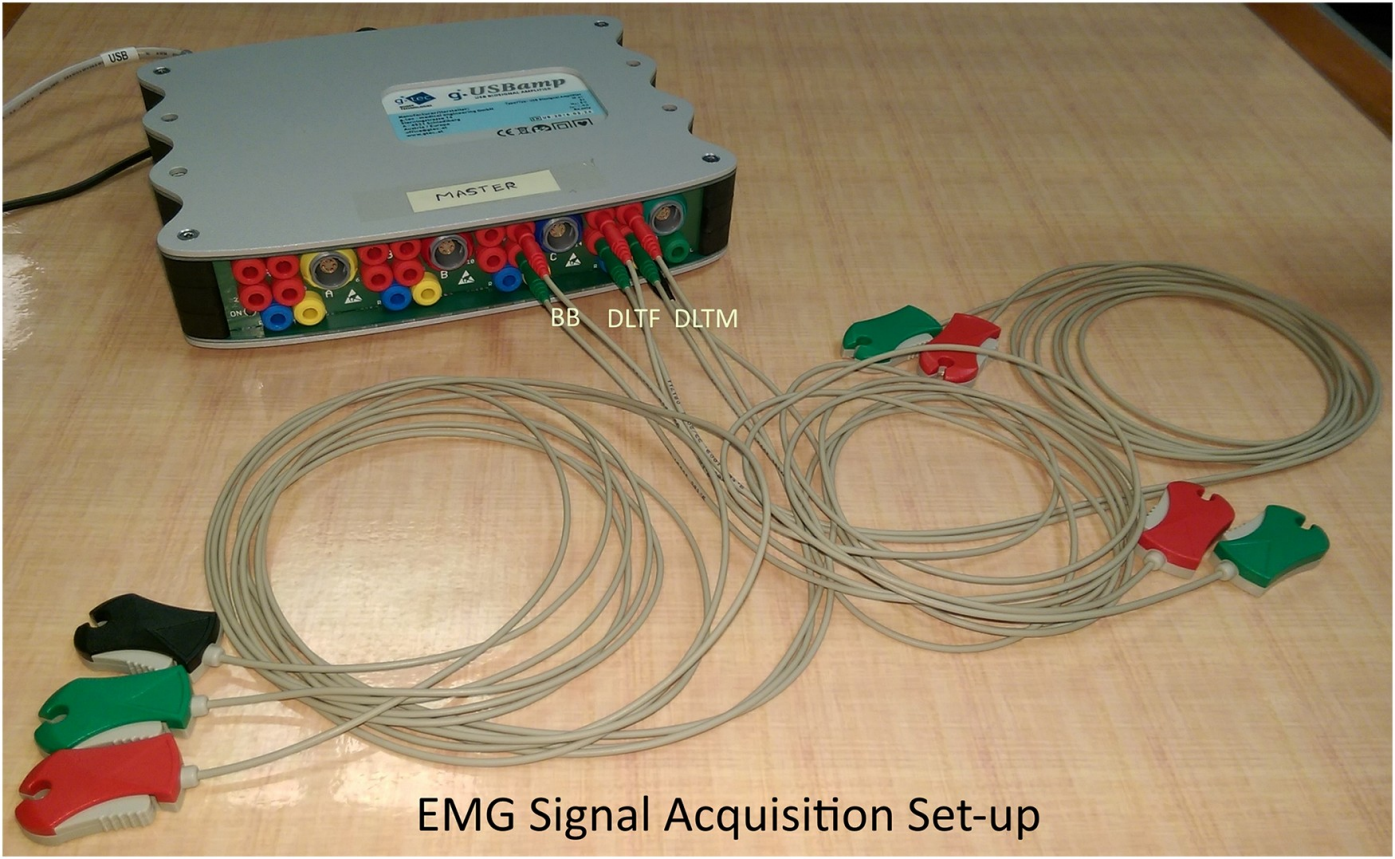
EMG signal acquisition setup. The EMG acquisition device with g.USBamp amplifier, three bipolar electrodes and a ground electrode.

**Fig 4:**
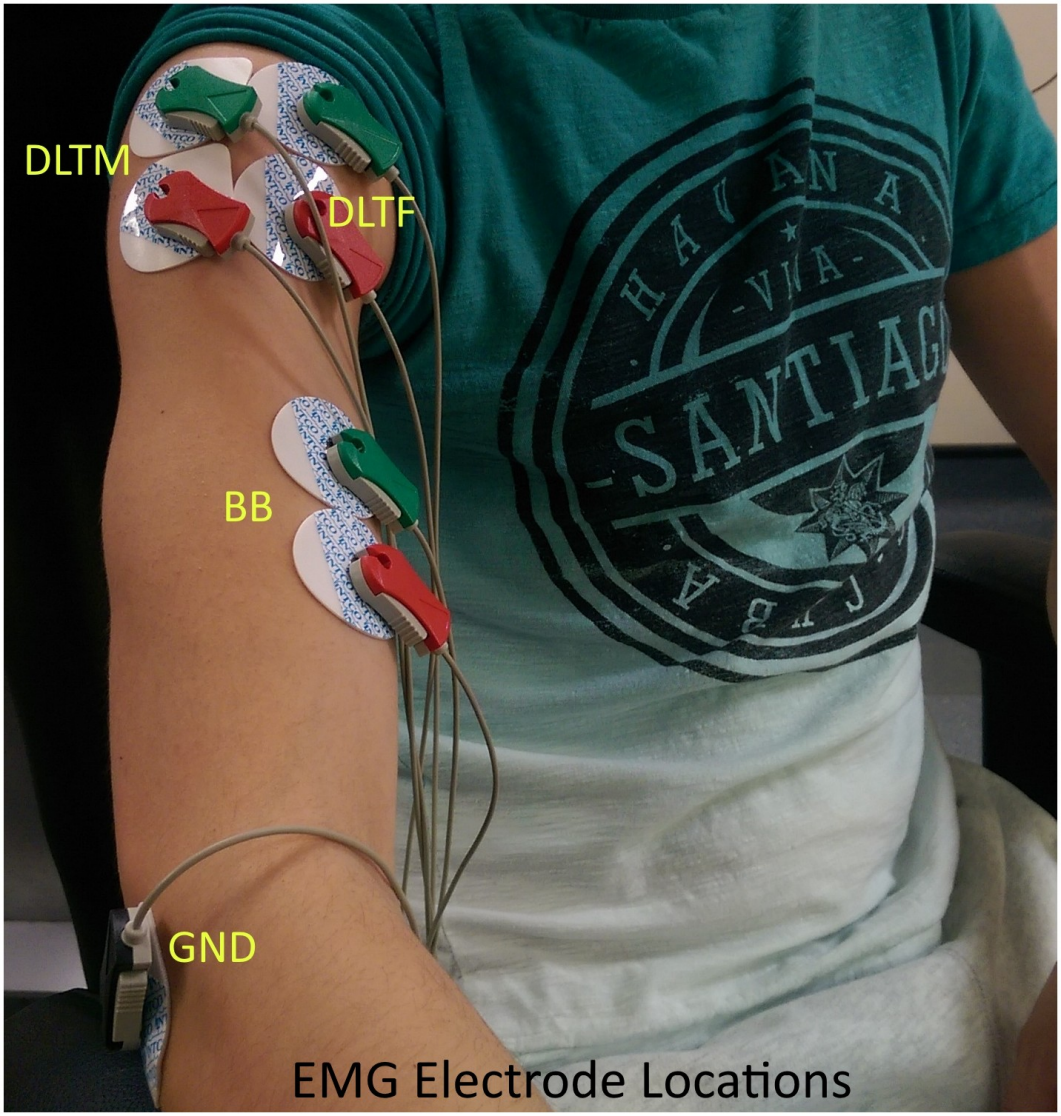
EMG electrode locations. EMG electrodes are connected to three upper limb muscle locations (Biceps Brachii (BB), Anterior Deltoid (DLTF), and Middle Deltoid (DLTM)). The ground electrode was connected to a bony area near the elbow.

A non-invasive EMG acquisition device (CE marked g.USBamp) from g.tec medical engineering GmbH, was used to acquire the EMG signals from the upper limb muscles. The configuration parameters for g.USBamp amplifier for data acquisition (sampling rate, channel selection, frame length, and electrode configurations) was set using a Simulink model. Three EMG electrode channels were configured in bipolar mode with a sampling frequency of 1200Hz. They were attached to three upper limb muscles, biceps brachii (BB), anterior deltoid (DLTF), and middle deltoid (DLTM). An electrode cable with a clip-lead was attached to sterile disposable non-invasive electrodes to measure EMG signals, as shown in Figs 3 and 4. A bipolar electrode configuration was used for the EMG acquisition, and the electrode placement errors were minimised by following SENIAM guidelines [44]. Participant’s demographic data such as body weight, body mass index (BMI), visceral fat classification, skeletal muscle percentage, and body fat percentage were measured using OMRON digital weight scale. This data was used for studying its correlation with the task performance measures during the off-line analysis, as explained later.

### Experimental protocol and methods

The experiment was designed for performing an upper limb exercise that simulates boat rowing using a robotic arm. The study compared how the change in task difficulty levels was perceived by the participants when the robot adjusted the difficulty when the difficulty was manually adjusted as well as when there was no difficulty adjustment at all. Three experimental conditions were chosen, one benefiting from robotic adaptation (the intervention group) and the other two presenting control groups 1 and 2. Thirty (30) healthy right-handed participants (13 female and 17 male) of at least 18 years old (mean ± SD: 31.8 ± 10.6 years) and with no history of injury to the upper limb and back were involved in the experiment. They were students or staff members of the University of Hertfordshire or volunteers from outside the university. The participants were asked to hold the robotic end-effector using their right hand with an animated boat rowing environment on an LCD monitor in front. They were free to move the robot in any path for rowing on the right side of the boat. The number of rowing iterations and the time duration of the tasks were studied, when the task was not auto-adjusted according to user’s state of fatigue (control groups) as well as when using an adaptive algorithm for altering the difficulty (the intervention group). Ethics approval was obtained from the Health, Science, Engineering and Technology Ethics Committee, University of Hertfordshire (Protocol number: aCOM/PGR/UH/03221(1)). Written informed consent was obtained from all individual participants included in the study.

#### Protocol

**Fig 5:**
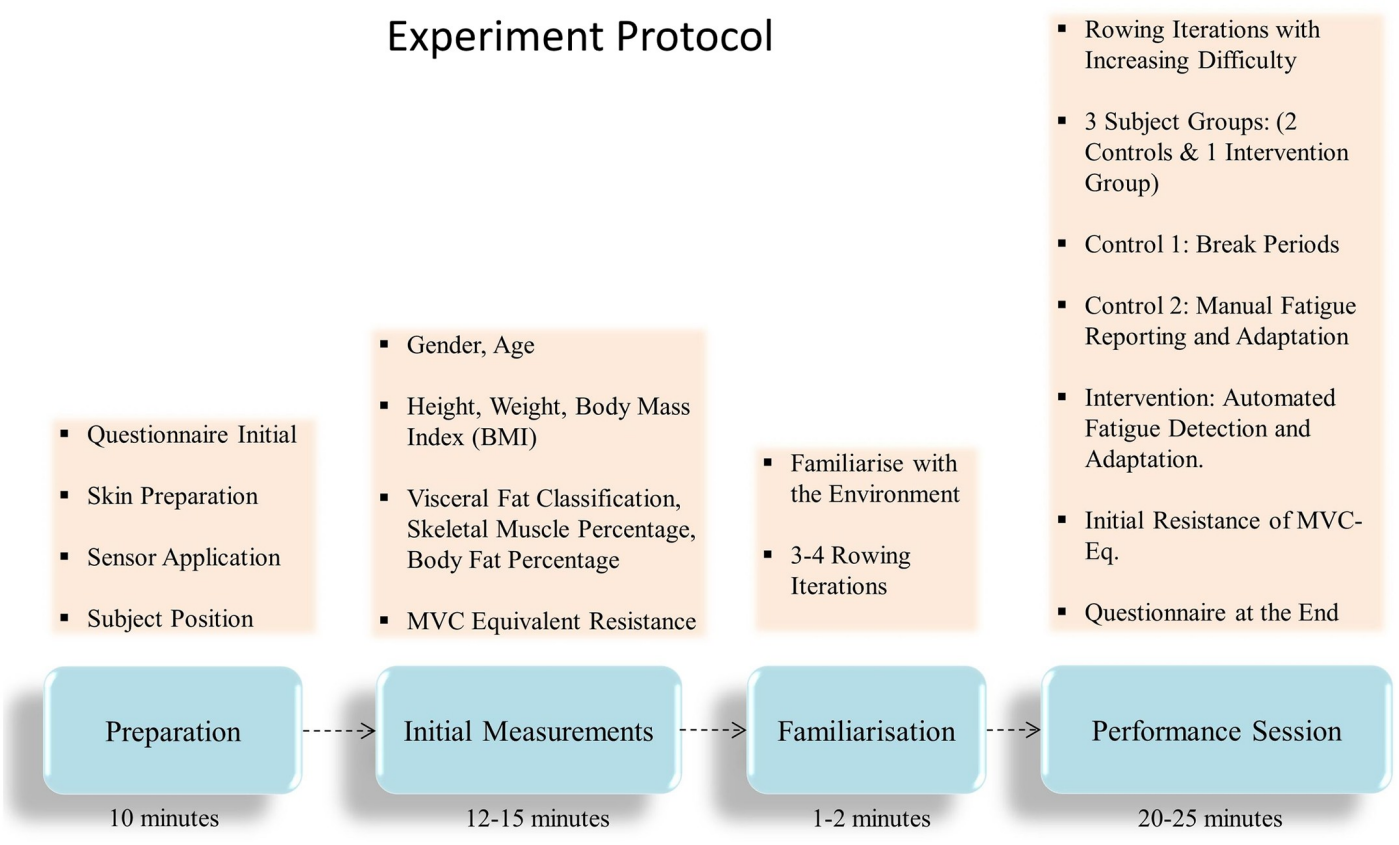
Experiment protocol. Description of the different stages of the experiment protocol.

The protocol for the current experiment was defined based on the study of Chang et al. [27]. Participants performed a set of maximum voluntary contraction (MVC) trials at first, then took a recovery break, followed with a low-intensity task, and then gradually increased the difficulty level. The protocol included a preparation stage, followed by initial measurements, a familiarisation session and finally a performance session (DOI: dx.doi.org/10.17504/protocols.io.5hrg356) as described in Fig 5.

The total duration of the experiment, including the setup time for each participant, was usually 40-55 minutes. Audio feedback was given regarding the start and end of the experiment cycles as well as during break periods. The participants were allowed to stop the session in case of any feeling of fatigue or discomfort during the experiment.

A questionnaire was given as part of the experiment to capture the subjective state of fatigue for each participant. The participants were requested to fill in a part of the questionnaire at the beginning and after the experiment. This collected various information such as the level of muscle fatigue before and after the experiment, the level of task difficulty, and their feeling about the effect of fatigue on their performance. The questionnaires also collected information such as, if they felt that the robot assisted them while doing the task if they received sufficient robotic assistance exactly when they needed, did the robotic assistance help them to perform more iterations, and which muscles were more tired. The answers were collected using a categorical scale. For example, the state of fatigue was collected using the categories, “Not Fatigued”, “Somewhat Fatigued”, “Fatigued”, “Very Fatigued”, and “Extremely Fatigued”. The level of task difficulty was categorised as “Too Easy”, “Somewhat Easy”, “Difficult”, “Very Difficult”, and “Extremely Difficult”. The collected information was later used during off-line analysis.

#### Preparation

EMG sensor electrodes were connected to the skin on the upper limb locations. The skin area for the sensor application was prepared by cleaning with a wet wipe. Participants were assisted to fix the EMG electrodes on the upper limb muscles. The ground electrode was connected to a bony area near the elbow, as shown in Fig 4. The participants were asked to sit straight on a non-rotating chair during the experiment. The hand involved in the experiment was not externally supported. Participants were advised to wear a loose garment for the ease of fixing the electrodes on the upper limb.

#### Initial measurements

Since the rowing task is of a dynamic nature, each participant will apply different levels of force according to their physical strength and the current state of fatigue. This will make it difficult to compare the task performance measures (time-to-fatigue, experiment duration, and the number of task repetitions) across the subjects. Also, the robot’s maximum force is not comparable to the range of maximum force variations in an average healthy individual. So, a personalised assessment was used to identify the maximum feasible force. Since the fatigue was planned to be produced through the resistance offered by the robot, it was decided to make the measurements of an isometric quantifiable nature by standardising the applied force/resistance across the different participants. Hence, the most feasible and highest robotic resistance for the rowing task was measured for each participant. The initial robotic resistance for each participant was set to a comfortable highest level by manually adjusting the damping coefficient of the robotic end-effector. Verbal feedback about the task difficulty was collected from each subject. The value of the damping coefficient corresponding to the highest feasible force/resistance was noted. Three such maximum voluntary contractions (MVC) were conducted. There was a break period of at least 30 seconds between each MVC trial [45, 46, 47]. The average value of the three readings was calculated [27]. This was termed MVC-Equivalent (MVC-Eq) and was approximated as each subject’s MVC force, which was used to set the initial task difficulty for each subject. After the three MVC trials, there was a break period for 10 minutes or until a self-reported full recovery before starting the rowing task [27]. The parameter MVC-Eq here does not actually represent force; instead it represents the damping coefficient that corresponds to the maximum feasible robotic resistance. So the MVC-Eq used in this study has a unit of damping coefficient (Ns/m) and not that of force. Hence, the MVC-Eq here is related to the damping force (force opposing the movement) as shown in Eq 1 [48]. The damping force is related to the velocity of movement as in the equation, and thus also to the rowing frequency adopted by different participants.

$$\begin{array}{c} Force_{damping}=DampingCoefficient\times Velocity \end{array}$$ (1)

The EMG measurements were taken from the three upper limb muscles of the participants. Demographic data of each participant was measured before the experiment. The gender, age, and height of the participants were also measured. Different parameters like subject group name, MVC-Equivalent, number of rowing iterations, kinematic measurements such as the end-effector position, velocity and force were logged into a file at a rate of 10 samples per second approximately. The different states of muscle fatigue were also logged during the experiment (reported fatigue, detected fatigue and relaxed state).

#### Familiarisation and performance sessions

After the initial measurements were completed, all the participants familiarised themselves with the robot and the environment in a practice run, which was then followed by the performance session. There were three experimental conditions and hence, three groups of participants in this study. They were randomly assigned to a group, and each group was assigned 10 participants each as described in Table 1. The intervention group was designed to involve the participants in a fatigue-adaptive robotic environment, where the subjects would receive varying resistance from the robot automatically based on their muscular state (fatigue) detected using EMG features. Control Group 1 was meant for studying the performance of the subjects in a similar environment as in the intervention group, but instead of receiving a robotic adaptation they were given break periods at regular intervals, and then an increased resistance after the break. They did not receive any adaptation from the robot during the interaction. Control Group 2 was designed for exploring the task performance during the same adaptive robotic environment as in the intervention group, but instead of receiving an automatic adaptation, the subjective state of muscular fatigue was used for the robotic adaptation. During these three experimental conditions, participants were asked to perform the exercise until they were unable to continue or until they reported fatigue three times or until the maximum feasible robotic resistance was reached.

**Table 1:** Participant table with gender, age and experiment groups. Each participant was assigned to different groups (Control 1, Control 2, and Intervention) randomly. There were 17 male and 13 female participants of at least 18 years old.

Groups	Age (mean ± std) in Years	Number of Male Subjects	Number of Female Subjects
Intervention	29.5 ± 8.17	5	5
Control 2	34.5 ± 14	6	4
Control 1	31.3 ± 9.36	6	4

During the performance session, the three groups of participants involved in each experiment branch received incremental robotic resistance at some intervals (1 minute). The experiment started at a low difficulty level of 20% maximum feasible MVC equivalent (MVC-Eq) resistance [49], then this was progressively incremented by 10% MVC-Eq in each trial, and then normally continued until the robotic resistance reached 100% MVC-Eq [27]. The part, where the current difficulty was incremented by 10% MVC was termed MVC+ part. However, there were different strategies for the break period or reducing the difficulty level in the three subject groups. The subjects were requested to report fatigue when they were in a state of pain or unable to continue, which helped to assess their psychological perception of fatigue. Participants of all groups were allowed to report fatigue orally during the interaction, which was then recorded as a subjective measure of fatigue. The experiment would continue until the participants reported fatigue three times or until the robotic resistance reached 100% of MVC-Eq. The maximum duration of the rowing tasks was set to 20 minutes, after which the experiment was manually stopped in order not to exceed the maximum experiment duration of 45-60 minutes.

#### Control-1 group

In Control-1 group participants, the protocol suggested by Chang et al. [27] of increasing difficulty for muscle training, with break periods in between each trial was implemented. The participants did not receive any adaptation from the robot, and there were no reducing of difficulty levels during the robotic interaction. While the status of the muscle fatigue was recorded, there was no intervention based on the detection of fatigue; instead, the participants could only report fatigue. The participants were asked to perform each trial for 1 minute [27] or until they felt tired before they could take a break of 30 seconds [50, 51, 52]. After each break, the next trial lasted for 1 minute. After the break period, the robotic resistance was incremented by 10% MVC-Eq before the subsequent trial. During the break period, the EMG acquisition was stopped, and the measurements were saved to a file.

#### Intervention group

In the Intervention group the participants interacted with an adaptive robotic environment, which was designed to adjust the difficulty level of the exercise automatically based on EMG fatigue indicators from the upper limb muscles. In addition to automatic fatigue detection, the participants were also allowed to report fatigue when needed. However, the reported state of fatigue was only saved to a file and not used for any adaptation. There were no break periods given; instead, there was a single continuous trial that incremented the difficulty level by 10% MVC-Eq every 1 minute. When the algorithm detected fatigue, the difficulty level was automatically decreased to 50% of the current value. The action when the value that caused fatigue was reduced to half and repeated was termed ‘reduce difficulty’ action. When relaxed, the robotic resistance was again incremented by 10% MVC-Eq after 1-minute trial, and then the trials were repeated until the resistance reached 100% MVC-Eq.

#### Control-2 group

There may be a delay in reporting fatigue compared to the fatigue that can be detected using EMG. This was studied by recruiting Control-2 group participants, where each participant reported the subjective fatigue, and the robotic adaptation was performed based on this. Similar to the Intervention group, there were no break periods between trials; instead, there was a single continuous trial that incremented the difficulty level by 10% MVC-Eq after every 1-minute trial. The robotic resistance was adapted only based on subject-reported fatigue and did not use the status of automatically detected fatigue. When the fatigue was reported by the participant, the difficulty level was decreased to 50% of the current value. After 1 minute, the robotic resistance started incrementing by 10% MVC-Eq, and then the trials were repeated until the resistance reached 100% MVC-Eq.

#### EMG data processing

**Fig 6:**
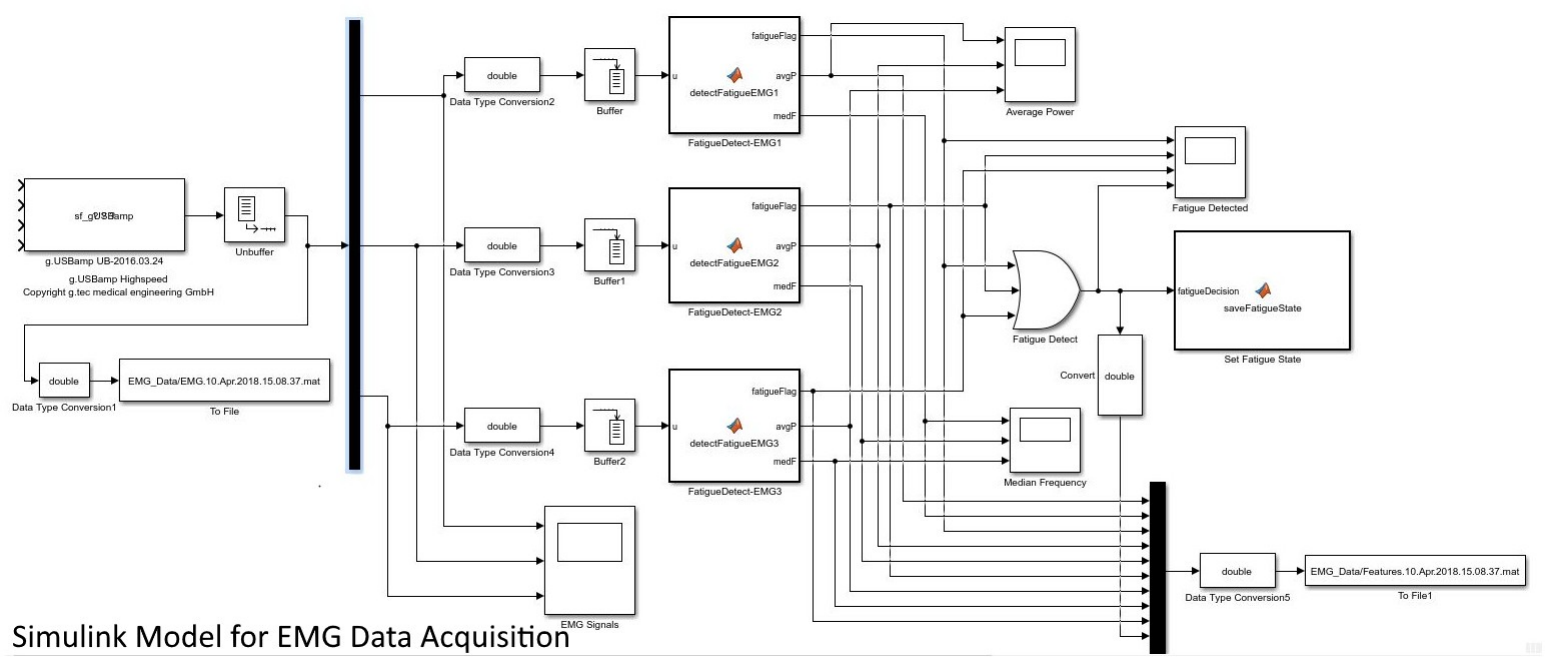
Simulink model for EMG data acquisition. The model has an on-line signal processing algorithm that performs fatigue detection for each muscle simultaneously. The detected fatigue state was communicated to the HapticMaster control algorithm.

The main part of the algorithm was implemented in an on-line modality. MATLAB 2016b was used to develop the EMG acquisition and on-line signal processing algorithms in Simulink. The g.USBamp hardware was supported by a data acquisition toolbox and used by downloading an adaptor API for the hardware. The hardware could be used from Simulink using the toolbox to incorporate live data into the Simulink model. Further analysis of the EMG, such as time-to-fatigue measurement and correlation study, were performed off-line. A de-multiplexer block was used to separate the individual EMG data from each electrode (Fig 6). Then, for each EMG channel, Simulink buffers were used to split the individual EMG data into blocks of fixed sizes. The average time taken to complete a single iteration rowing task was around 6 seconds. Hence, a buffer size of 7200 samples was used in the model (an average of 6 seconds per iteration x a sampling rate of 1200) to split the EMG into blocks. This buffer size was then used as the window length for EMG processing and analysis. MATLAB Function blocks were used to develop custom functions that processed the EMG data frames from the three muscles simultaneously and generated a fatigue status from all the three muscles separately. These function blocks calculated the EMG features (median frequency and average power) corresponding to one buffer length of the EMG from three muscles, as shown in Fig 6. Within each function block, a notch filter was used at 50Hz to remove the power line interference. For calculating the average power, the EMG was band-pass filtered at 0.8-2.5Hz [28], and for median frequency, at 20-450Hz. The MATLAB 2016b library function, “medfreq()” was used to estimate the median frequency of the power spectrum of EMG signal in terms of the sample rate. Average power was estimated using the “bandpower()” function provided by the MATLAB library. During the experiment, the EMG raw values and the estimated EMG features (average power and median power frequency) were also logged into csv files.

The EMG features calculated from the initial EMG blocks for each muscle were also used to calculate a baseline range to be used for fatigue detection. This baseline feature range corresponded to a muscle state before any occurrence of fatigue during the exercise. The baseline range was later used as a threshold to compare with the features from the following EMG measurements as the exercise progressed. The first three frames (corresponding to the initial 15 seconds) were ignored to avoid random fluctuations at the beginning of the EMG data, which might result in a wrong calculation of the baseline range. The features corresponding to the next frames were saved into a feature array, and were used for the calculation of the baseline range as described in Eq 2 [53, 54, 55]. The mean and standard deviation (STD) of the first five elements of the feature array were calculated, which corresponded to the movements during the initial 30 seconds approximately, ignoring the skipped initial frames. The duration of 30 seconds was approximated based on the inferences during the pilot trials, which would not have had an instance of fatigue at the initial stages of the exercise.

UpperLimit=mean(x)+2*STD(x) (2)

LowerLimit=mean(x)−2*STD(x) (3)

where *x* was the input baseline array of features.

Past studies have utilised EMG amplitude and median power frequency as fatigue indicators in various contexts [35, 36, 37, 38, 39]. As per our previous study, a statistically significant increase in the average power and decrease in median frequency were identified as indicators of muscle fatigue during an assist-as-needed robotic interaction [31]. Hence, a 2-times standard deviation check based on Eq 2 was used to decide if a new value of EMG feature was within the range or outside the range. For each muscle, if the feature values were out of range three times continuously, then a corresponding fatigue flag was set. When the features were within the baseline range, the fatigue flag was cleared. For EMG median frequency, a significant decrease in its value would cause fatigue detection, if the current value is below the lower limit of baseline threshold three times consecutively. If the value increases back to the baseline range three times consecutively, then the corresponding muscle was considered relaxed.

It was noticed during the pilot studies that the MVC+ step added some additional force requirement on the muscles while performing the rowing task, which resulted in an increase in the EMG amplitude and, hence, also in the average power. In shared control, where impedance/admittance control methods are used for robot-assisted strength training, the exercise intensity was found to increase as the desired damping increased [56]. The muscle activation was linear and proportional to the resistive load. Also, when the damping coefficient goes high, the speed of the task would also decrease. Hence, the result of using a 2-STD check for fatigue detection using EMG average power can be an indication of both muscle fatigue and the higher force requirement due to the MVC+ increments. However, this does not seem to be the case for median frequency due to the different physiological reasons behind the shift in the frequency spectrum during fatigue [38, 39, 35]. Since the average power was always showing an increase due to the MVC+ increments, only the median frequency was used in the fatigue detection algorithm. This makes the method similar to the JASA method [57, 58]. However, the average power was also logged during the experiment for the off-line analysis.

#### Simulink model for fatigue detection

As shown in the Simulink model (Fig 6), processing of the EMG measurements from the three muscles and detecting the corresponding muscular fatigue were done in parallel. If any of the three muscles indicated fatigue, a corresponding fatigue flag was set to 1. The final state of the upper limb fatigue was indicated by a separate fatigue flag, and this was done based on a logical ‘OR’ operation on the three individual fatigue flags corresponding to each EMG electrode. The final state of fatigue was then communicated to the HapticMaster control software through a shared csv file. The fatigue status was checked by the robotic algorithm by reading this file approximately every 6 seconds corresponding to one buffer size of EMG data. Since the robotic adaptation was not too time-critical, this way of communication was sufficient to feedback the state of muscles. If the robotic assistance had helped to reduce the fatigue, the muscle activation was supposed to be back to the normal range (within the baseline range). The fatigue flags were reset by the algorithm when the feature values returned to their baseline range. The individual muscles were considered relaxed when the corresponding fatigue flags were reset to zero. The upper limb was considered relaxed only when all the individual muscles were identified as relaxed. The relaxed state of the muscles was then updated in the shared csv file accordingly, which would inform the robot that the upper limb muscles were relaxed.

#### Adaptive robotic algorithm

**Fig 7:**
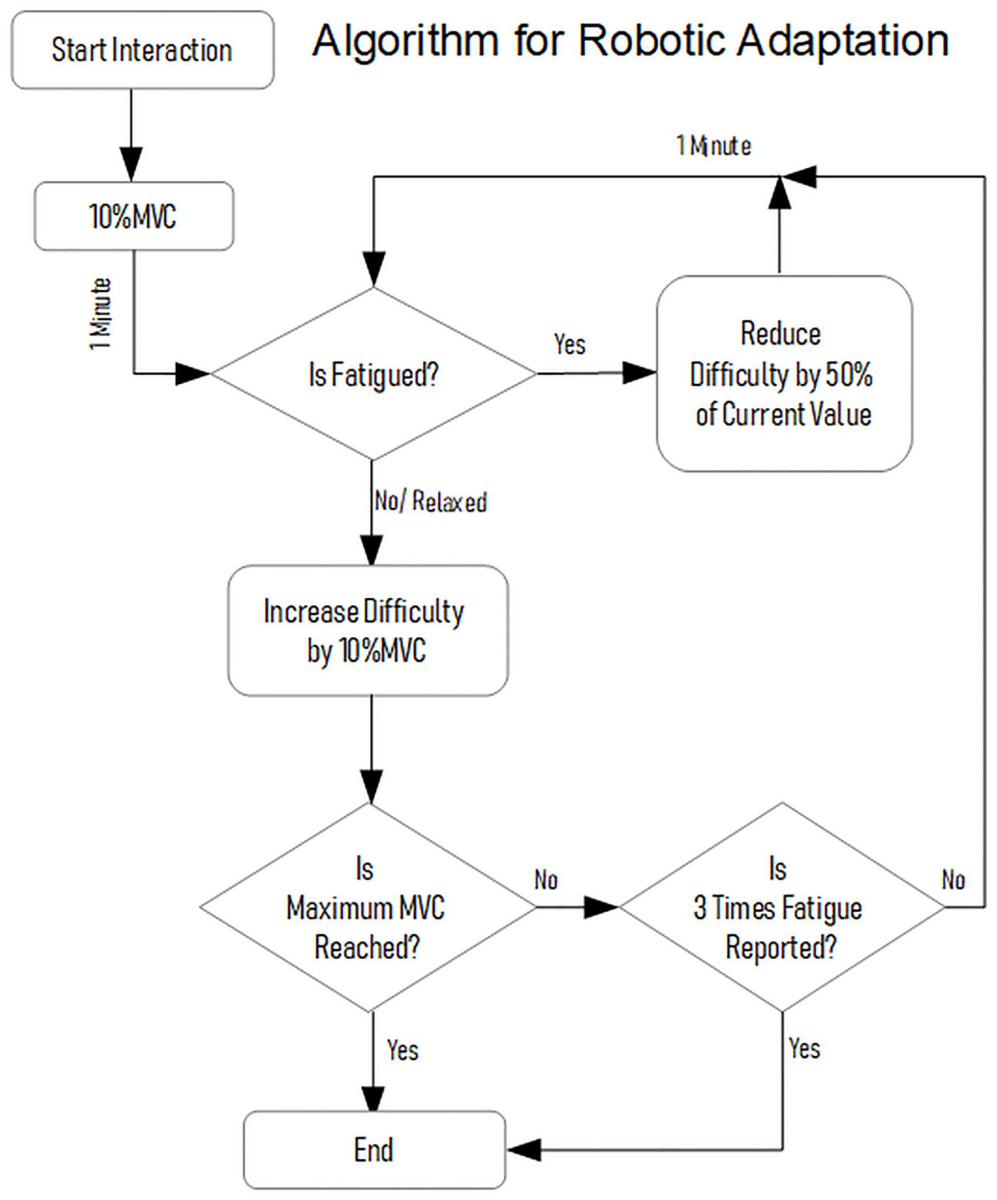
Algorithm for robotic adaptation. The flow chart of the adaptation algorithm for Intervention group participants.

The adaptive robotic algorithm was designed to support the intervention group participants when fatigue was detected based on the changes in the EMG features. The fatigue flag was checked by the robotic algorithm to adapt its behaviour accordingly. As explained in the protocol (Fig 7), after detecting fatigue, the robotic resistance was reduced to 50% of the present value that caused the fatigue. The ‘reduce difficulty’ action continued as long as the fatigue flag was set. Once the difficulty was reduced by 50%, the next ‘reduce difficulty’ action would only happen after the current trial of 1-minute duration was completed or after the muscles are relaxed once. Hence, if a fatigued state continued even after receiving the ‘reduce difficulty’ action earlier, the difficulty would further reduce by 50% in the next trial. This would go on until all the muscles were relaxed. Since there was a possibility that one of the muscles get fatigued, and then never relaxed, the assistance algorithm had to make sure that all the muscles were relaxed. This was done by the robot by providing additional anti-gravity support temporarily when the difficulty level reached the lowest value. This support was withdrawn once the muscles were relaxed at the lowest level of difficulty, and the MVC+ increments started again.

#### Off-line data analysis

An off-line data analysis was conducted on the different parameters calculated/obtained from the experiment. The parameters such as the time taken to automatically detect fatigue, the time of reported fatigue, the variation in the difficulty levels, the total duration of the experiment, the number of task repetitions/iterations, and the repetitions per minute were studied. For Control-1 group participants, the time-to-fatigue was calculated off-line from the EMG features, since there was a break period between different trials (of 1-minute duration), and the EMG data acquisition was stopped after each trial. The continuous progress of the EMG fatigue indicators could not be understood directly from the individual trials. Hence, the individual mat files containing the EMG data from each trial were merged to form a single combined mat file for each subject. The combined data was then analysed to detect fatigue as the trials progressed. However, some unexpected signal spikes were observed at the beginning of each trial after each break period when the device started capturing the EMG data. This appeared as an unexpected increase of EMG amplitude and, hence, resulted in a wrong fatigue detection by the algorithm. Hence, during the off-line EMG analysis, while combining the different mat files corresponding to each EMG acquisition, the initial spikes were avoided by ignoring the initial second of EMG data from each trial. Additionally, after combining the files, the initial 12 seconds of combined EMG data corresponding to the first two frames were also skipped during the off-line processing. However, during the on-line EMG processing for the Intervention and Control 2 groups, there was one additional empty frame of data at the beginning that was generated by the Simulink buffer block, and this occurred when the model started running. Hence, this additional frame was also skipped during the on-line processing, which made the total number of skipped initial frames 3. But in off-line processing, this was not required since this additional empty frame did not exist, and hence, only two frames were skipped.

During the analysis, the time-to-fatigue for the Intervention group and Control-2 group participants were calculated based on the data read from the HapticMaster log file using an algorithm developed in Python 3.7. The log file contained different information like the timestamps, end-effector position, velocity, and force, detected and reported-state of fatigue, iterations, and task difficulty level. The time-stamp corresponding to the state of fatigue was noted and used to calculate the time-to-fatigue. However, for Control-1 group participants, the time-to-fatigue was measured from the values of fatigue detection flags set by the off-line algorithm in MATLAB and by noting the corresponding window numbers. The corresponding window numbers were then multiplied by the window length to get the corresponding time-to-fatigue. Since the kinematic data corresponding to the break period between different MVC+ trials was also ignored, the time duration of the exercise in Control-1 group was calculated after skipping these periods. The number of task repetitions and the speed of repetitions (per minute) [42] were also analysed. A comparison was also made between the results considering the 2-STD threshold against 3-STD, 4-STD, and 5-STD thresholds in the algorithm for fatigue detection.

A correlation study was conducted between the participant demographic data (BMI, body fat percentage and skeletal muscle percentage) and the task performance measures (time-to-fatigue, experiment duration, and the number of task repetitions). This was performed to see if there is any influence of the demographic data on muscle fatigue, even after standardising the initial task difficulty based on the MVC-Equivalent for each participant. Spearman’s correlation analysis was performed using IBM SPSS v22.

## Results

### Median frequency analysis

Similar to the results from the previous experiments [31], an increase in average power and a decrease of median frequency were observed and confirmed during the analysis. In the current study, a decrease in median frequency was never accompanied by a decrease in the amplitude as verified during the off-line analysis. Hence, a decrease in muscle force was not the probable cause for the above response of the EMG features; instead, it was the muscle fatigue which caused this.

**Fig 8:**
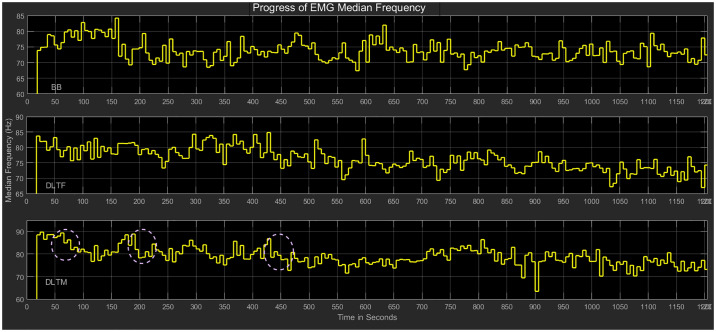
Progress of EMG median frequency. Median frequency in a typical Intervention group participant (Subject 20), who received adaptive robotic assistance based on the detected muscle fatigue using EMG features. The doted regions represent a significant decrease in median frequency, which resulted in the detection of fatigue.

**Fig 9:**
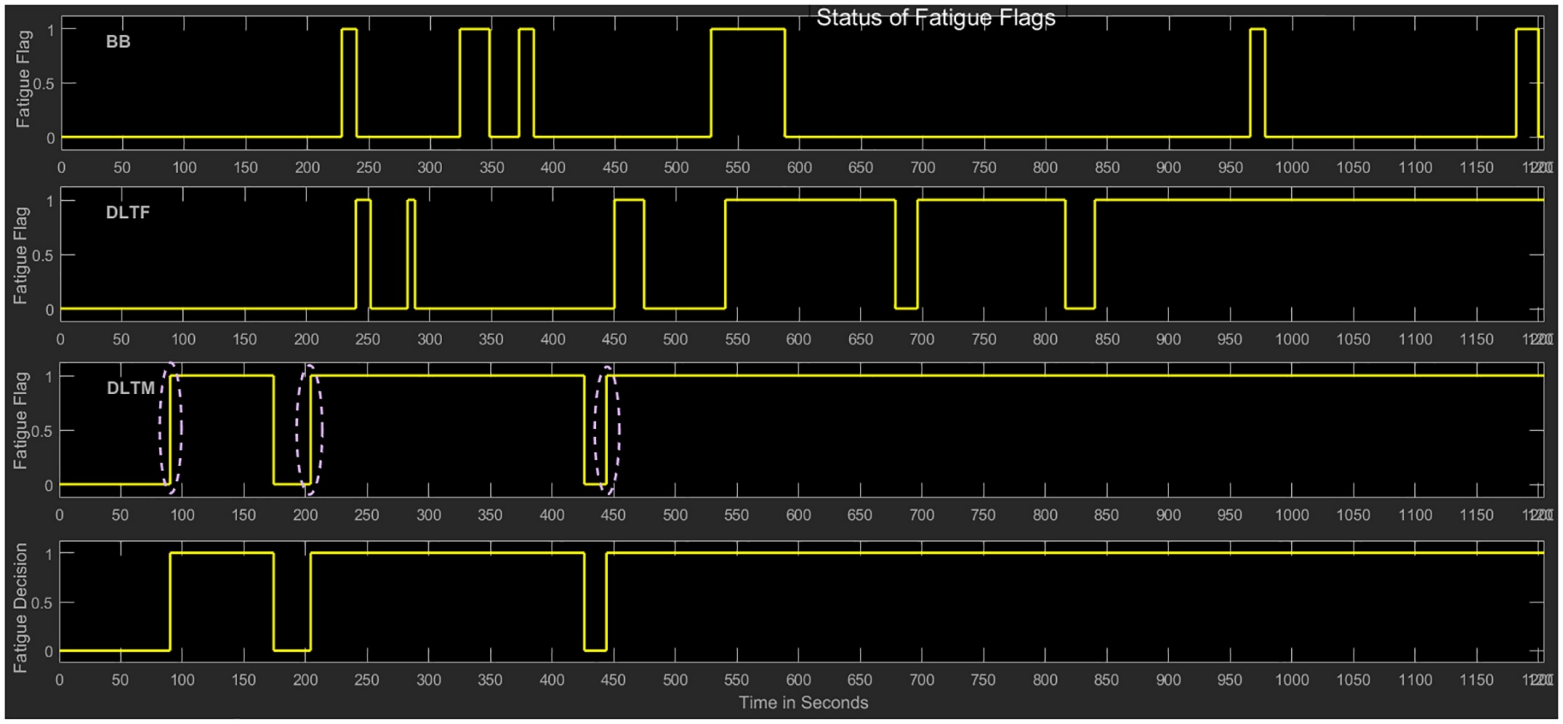
Status of fatigue flags. The fatigue flags in a typical Intervention group participant (Subject 20), who received adaptive robotic assistance based on the detected muscle fatigue. The doted regions represent the detection of fatigue in the DLTM muscle, which decided the final state of fatigue.

The variation of median frequency over time for a typical subject and the corresponding detection of fatigue in the individual muscles (BB, DLTF, DLTM) are shown in Figs 8 and 9, respectively. The method for fatigue detection was based on our previous work on fatigue detection in a robotic environment [31]. Feature line plots that show the progress of EMG features as the different muscles were fatigued and relaxed during the adaptive robotic interaction are shown in Fig 8. The median frequency in the Intervention group participant displayed both increases and decreases based on the robotic adaptation. The doted regions indicated a significant decrease of median frequency in the DLTM muscle, which caused in the detection of fatigue. The initial values showed that the muscle fatigue was detected when the EMG feature went outside the 2-STD threshold range and relaxed when the feature was back in the normal range. For example, for a typical subject in Intervention group the baseline range (using 2-STD threshold) for BB muscle was calculated using the initial five values of median frequency in Hz (73.87, 74.86, 74.92, 78.92, and 78.52), which resulted in a range of 80.87 Hz to 71.56 Hz. Muscle fatigue was later detected when the feature values went below this range three times continuously (70.73 Hz, 69.40 Hz, 71.48 Hz) as the time progressed. The final fatigue flag was set based on the individual fatigue flags for each muscle. The doted regions indicated the detection of fatigue in the DLTM muscle, which decided the final state of fatigue in this case, as shown in Fig 9. The other muscles, even though underwent many cycles of fatigue and relaxation, were not the deciding factors in the final decision about fatigue.

### Robotic resistance adaptation

**Fig 10:**
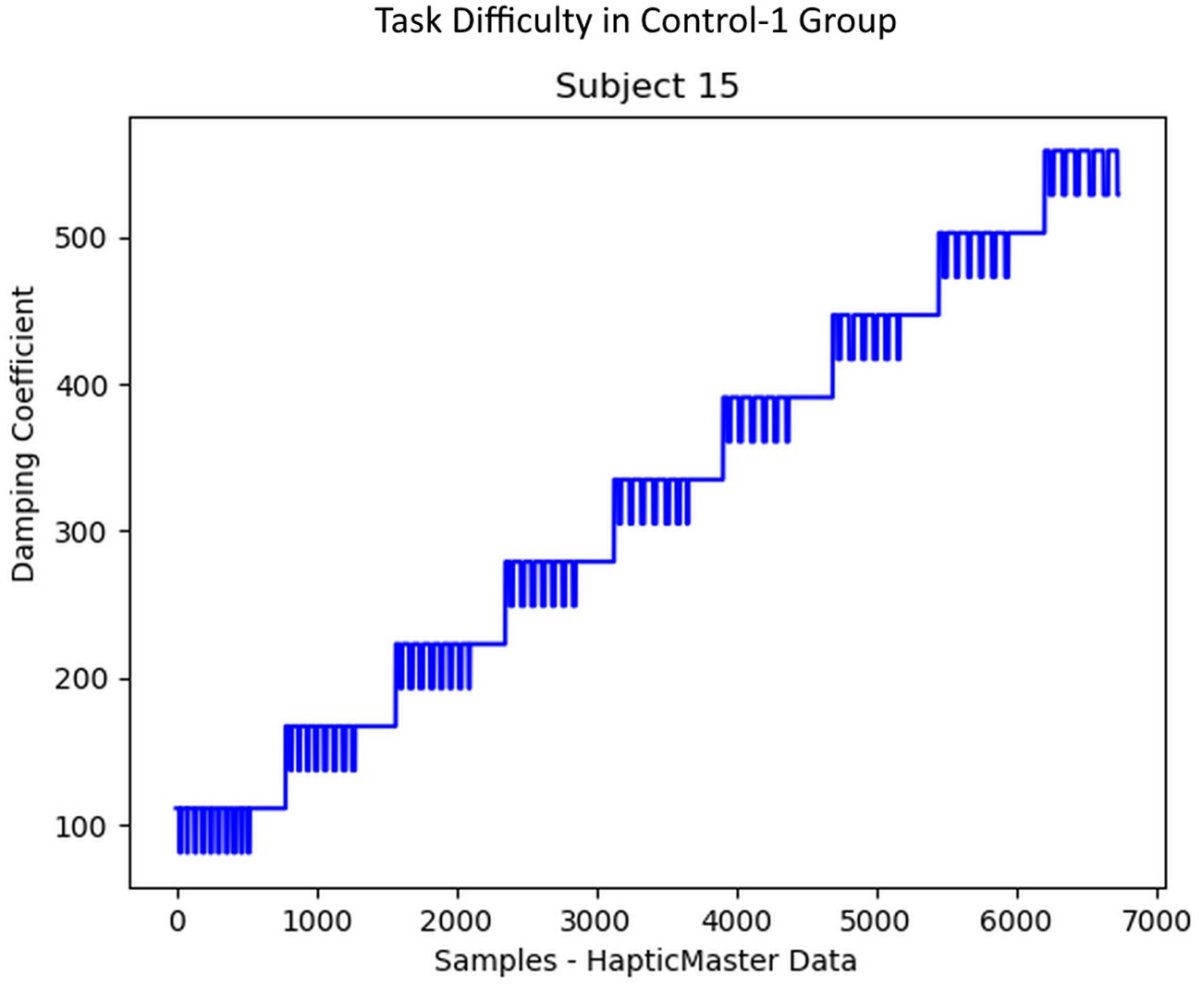
Task difficulty in Control-1 group. The progress of task difficulty in Control-1 participants, who received 30 seconds break period after each trial of 1-minute duration before the MVC+ increment. This group did not receive any robotic adaptation based on muscle fatigue.

**Fig 11:**
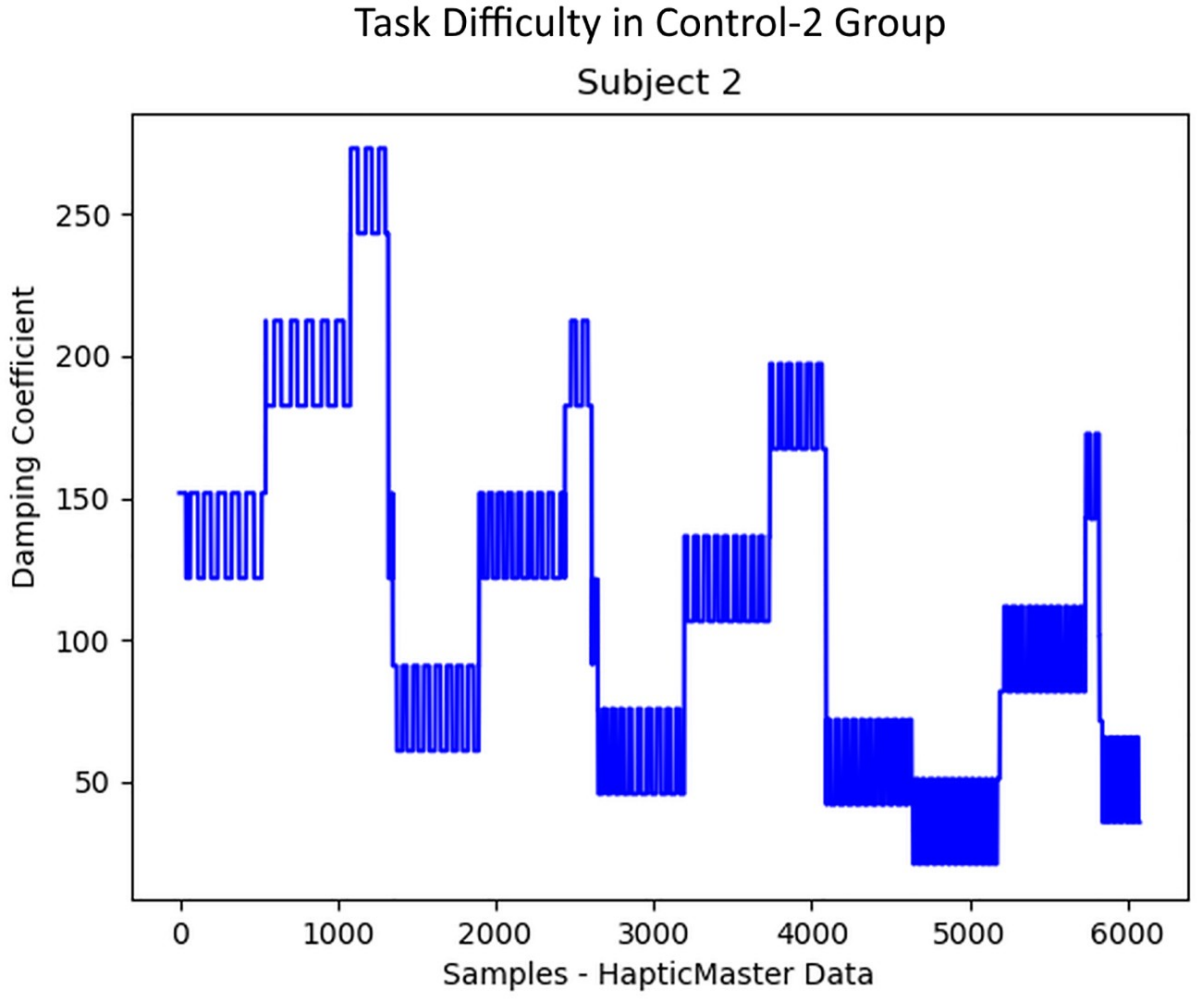
Task difficulty in Control-2 group. The progress of task difficulty in Control-2 group participants, who received a manual robotic adaptation based on the subjective fatigue reported.

**Fig 12:**
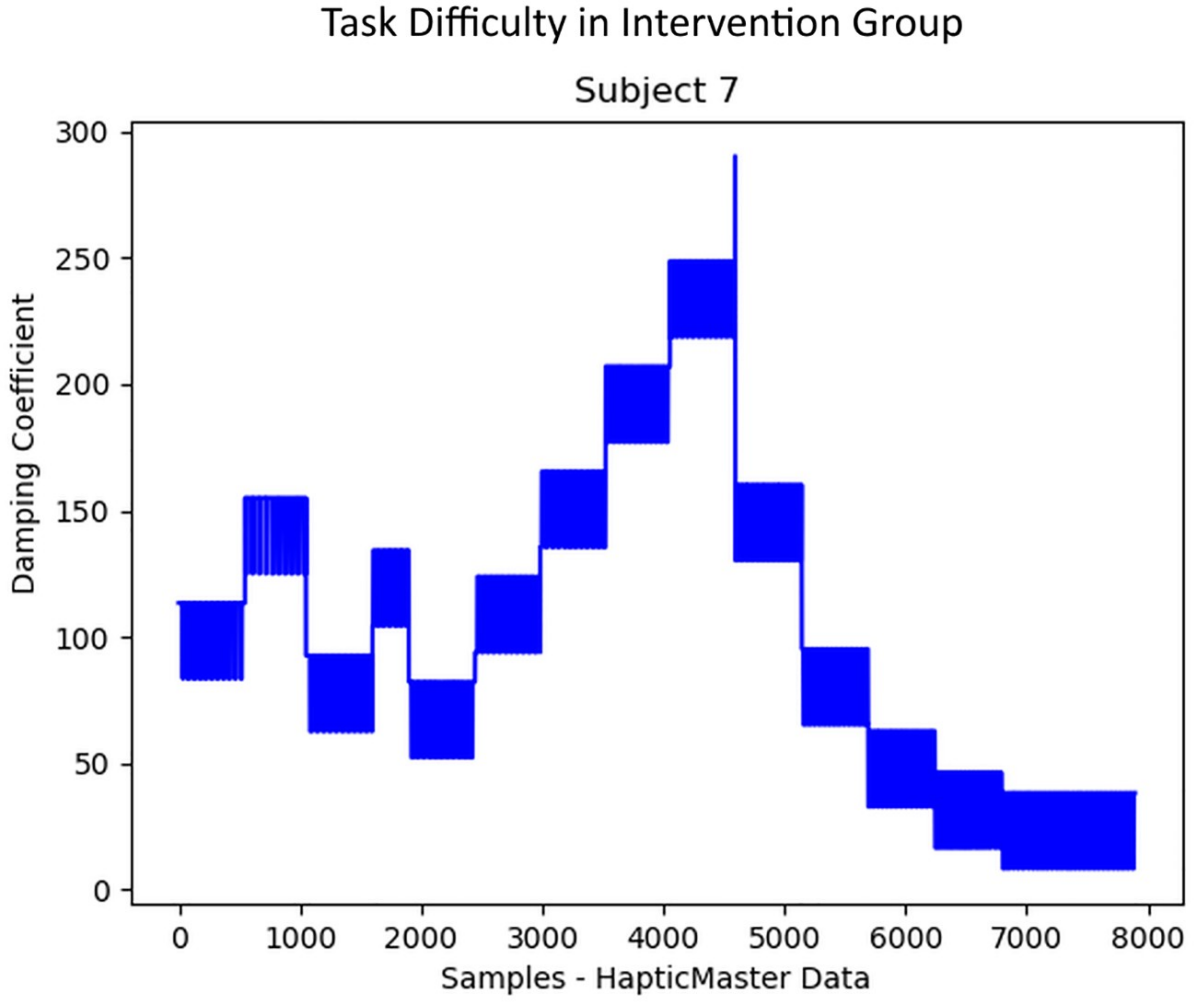
Task difficulty in Intervention group. The progress of task difficulty in Intervention group participants who received an automatic robotic adaptation based on the detected fatigue using EMG features.

The progress of the difficulty levels/damping coefficients during the rowing task for the three groups of participants was plotted, as in Figs 10, 11 and 12. The Control-1 group performed the experiment with difficulty taking much effort to complete, however, was not able to do many iterations due to the progressively increasing difficulty after every 30 seconds break period as shown in Fig 10. After the experiment, only two subjects in the Control-1 group reported fatigue, four subjects reported “somewhat fatigued”, and the remaining four reported “not fatigued” through the final questionnaire. The break period introduced to avoid fatigue seemed to have helped many of them to recover before each trial. In Control-2 group participants, a subjective reporting of fatigue during the experiment was used to manually reduce the task difficulty by 50% of the current value as shown in Fig 11, which helped them to prolong the exercise. When the subjects reported relaxed, the difficulty started increasing by 10% MVC, as shown in the figure. The questionnaire response after the experiment stated that five subjects reported “fatigued” and the remaining five reported “somewhat fatigued”. None of them reported “Not Fatigued”.

Intervention group participants could do many iterations of rowing task due to the auto-adaptation of the task difficulty based on their muscular state. Several of them could continue the experiment until the maximum duration of 20 minutes was reached. Many participants did not report fatigue during the session, and the experiment had to be stopped since the time limit was exceeded. However, as shown in the plot of difficulty levels in Fig 12, it was noticed that muscle fatigue was detected by the algorithm many times during the task, and the difficulty was automatically adjusted by the algorithm accordingly. The difficulty was reduced to 50% of the current value each time when the algorithm detected fatigue. When a relaxed state of muscles was detected by the algorithm, the participants received progressively challenging increments in the difficulty level by 10% MVC.

A non-parametric (Kruskal-Wallis) test on the damping coefficient across the three subject groups showed that there was a significant difference across them. The results show that the three groups explained 36.3% of the variability in the damping coefficient (robotic resistance). Among the three groups, the pair-wise comparison between Control 1-Intervention groups had the highest effect on the variability in the robotic resistance (41% of the variability). The median values of robotic resistance between the groups indicated that the Intervention group had the least median value compared to the other two groups, as shown in Table 2.

**Table 2:** Comparison of medians of damping coefficient across the three subject groups. Intervention group had the least value compared to Control 1 and Control 2 groups.

Comparison of Medians (of Damping Coefficient)			
**Control 1**	**N**	Valid	57477
		Missing	0
	**Median**		230.000000
	**Std. Deviation**		121.2779196
**Control 2**	**N**	Valid	51386
		Missing	0
	**Median**		144.800000
	**Std. Deviation**		93.9520811
**Intervention**	**N**	Valid	89091
		Missing	0
	**Median**		62.500000
	**Std. Deviation**		83.0499727

This showed that the automatic detection of fatigue based on EMG features by the algorithm resulted in a switching of the task difficulty. This helped the participants to avoid or delay a state of fatigue during the interaction and, hence, to have more repetitions and a prolonged robotic interaction.

The adaptive robotic algorithm produced the task difficulty as the output, and this was decided based on the muscular state of the participants. Analysing the average task difficulty offered by the adaptive algorithm for the different participant groups indicated that Control-1 group participants faced the greatest task difficulty. Since the Intervention group received auto-adaptation of the task difficulty during the progressively challenging task, their average task difficulty was significantly lesser than that of the control groups.

### Task performance measures

Task performance was measured using the analysis of variance (ANOVA) method for the different features as in Table 3.

**Table 3:** Analysis of variance (ANOVA)for the task performance measures.

ANOVA Table						
		Sum of Squares	df	Mean Square	F	Sig.
Number of Repetitions * Group	Between Groups (Combined)	129105.000	2	64552.500	14.792	0.000
	Within Groups	117829.000	27	4364.037		
	Total	246934.000	29			
Duration(s) * Group	Between Groups (Combined)	1489556.828	2	744778.414	19.093	0.000
	Within Groups	1053229.088	27	39008.485		
	Total	2542785.915	29			
Repetitions / min * Group	Between Groups (Combined)	61.476	2	30.738	2.794	0.079
	Within Groups	297.048	27	11.002		
	Total	358.523	29			
Time to Reported Fatigue (s) * Group	Between Groups (Combined)	985373.911	2	492686.956	15.778	0.000
	Within Groups	374716.097	12	31226.341		
	Total	1360090.008	14			

#### Task duration

**Fig 13:**
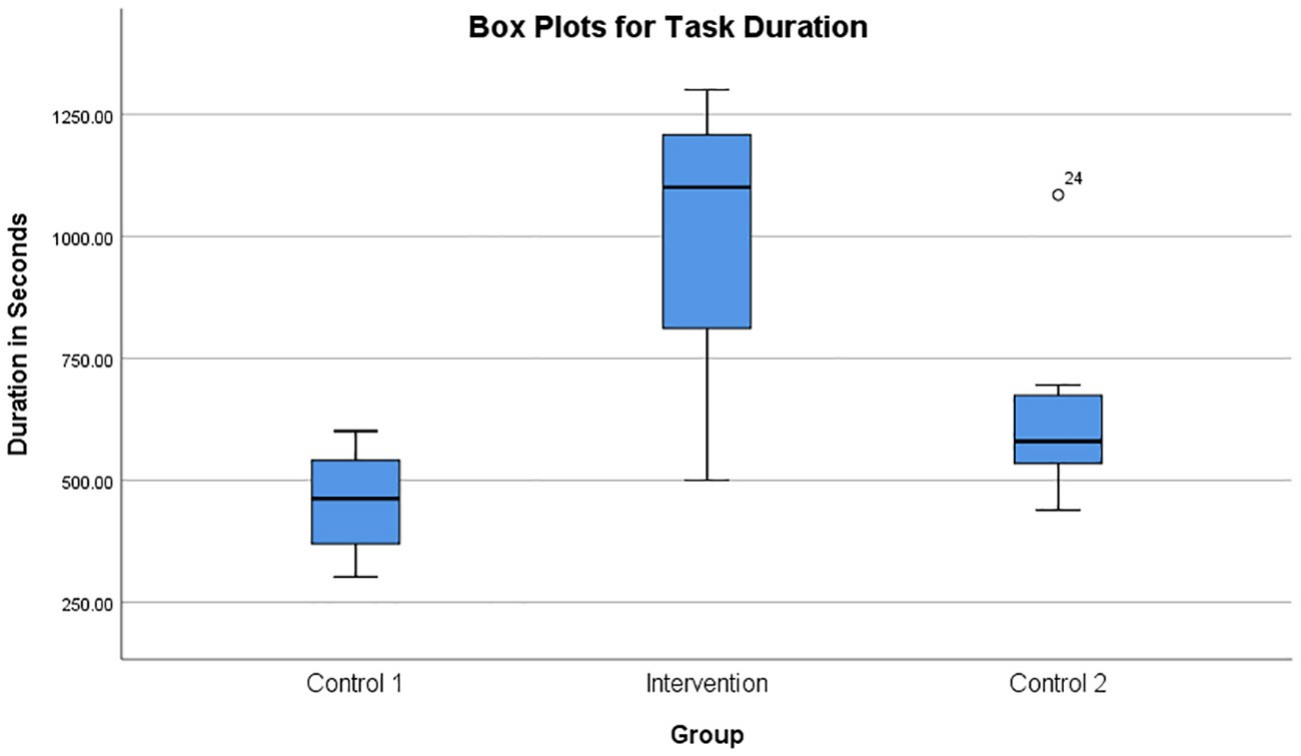
Box plots for task duration. Box plots showing the duration of the experiment in the three groups of participants.

Analysing SPSS box plots of the duration of the experiment in the three subject groups as shown in Fig 13 indicated that the Intervention group participants who received adaptation had the highest duration compared to Control-1 group who did not receive any adaptation. In the Control-1 group, the 30 seconds of break period between different trials were ignored while calculating the duration. The Control-2 group displayed a better duration compared to Control-1 group but less than that of the Intervention group participants. The analysis of variance (ANOVA) of task duration across the three subject groups also supported these results, as shown in Table 3. Using ANOVA, the Intervention group had a statistically significant (*F*(2, 27) = 19.093, *p* < 0.005) prolonged interaction due to the auto-adaptation of the task difficulty during the progressively challenging exercise.

#### Number of repetitions

**Fig 14:**
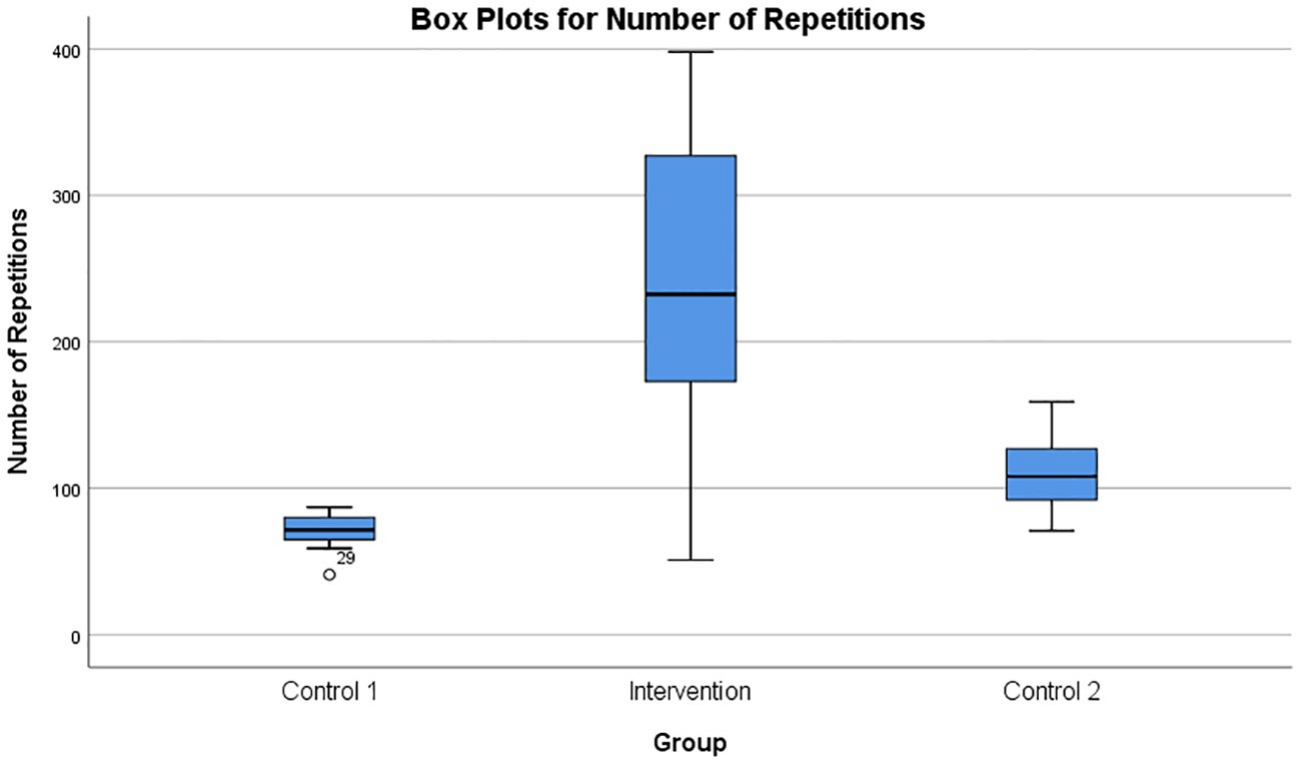
Box plots for number of repetitions. Box plots showing the number of repetitions of the rowing task in the three groups of participants. The Intervention group could do more task repetitions due to the auto-adaptation of the task difficulty during the progressively challenging exercise.

**Fig 15:**
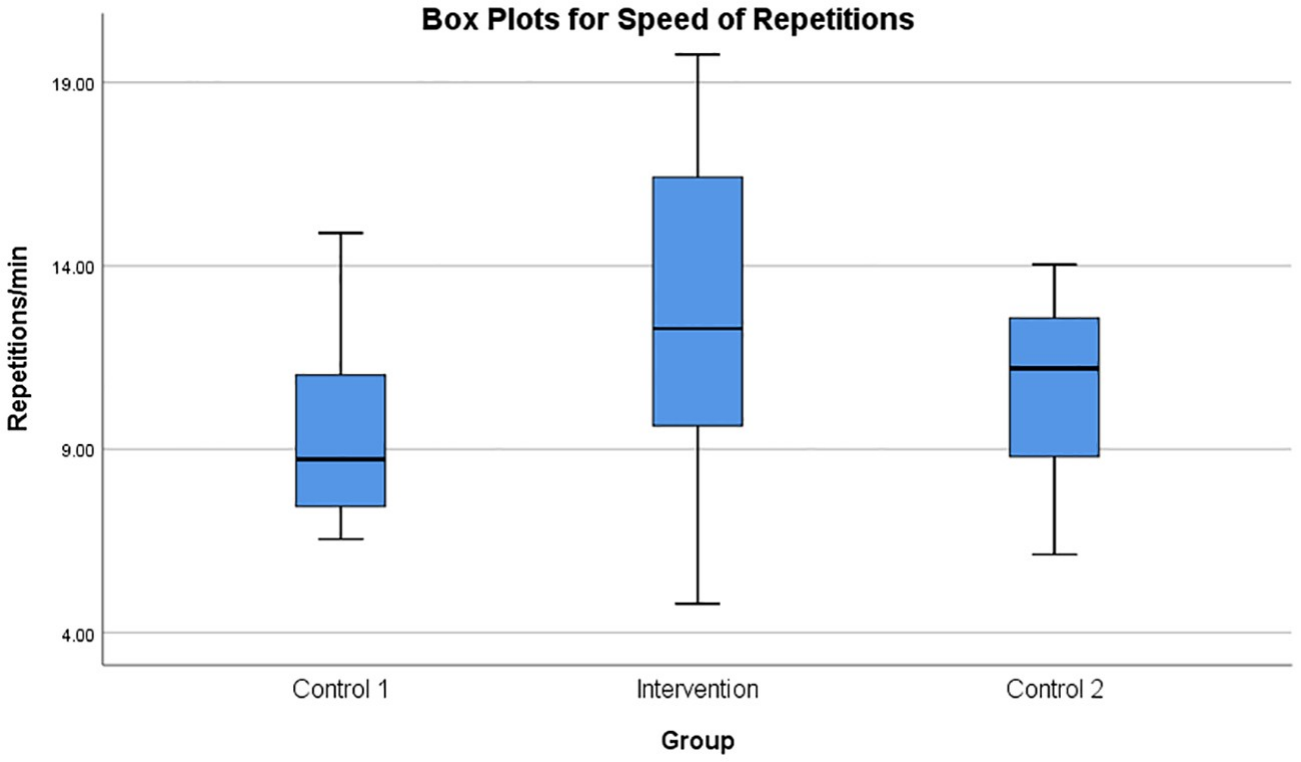
Box plots for speed of repetitions. Box plots showing the rate of task repetitions (repetitions/minute) of the rowing task in the three groups of participants.

The number of repetitions during the rowing task was compared using the box plots, as shown in Fig 14. There was a statistically significant difference in the number of repetitions in the three groups, where the Intervention group displayed the highest values compared to Control 1 and Control 2 groups (*F*(2, 27) = 14.792, *p* < 0.005) as shown in Table 3. The number of task repetitions per minute was also calculated and analysed as shown in Fig 15, presenting close to significant differences (*F*(2, 27) = 2.794, *p* = 0.079).

#### Time to fatigue

**Fig 16:**
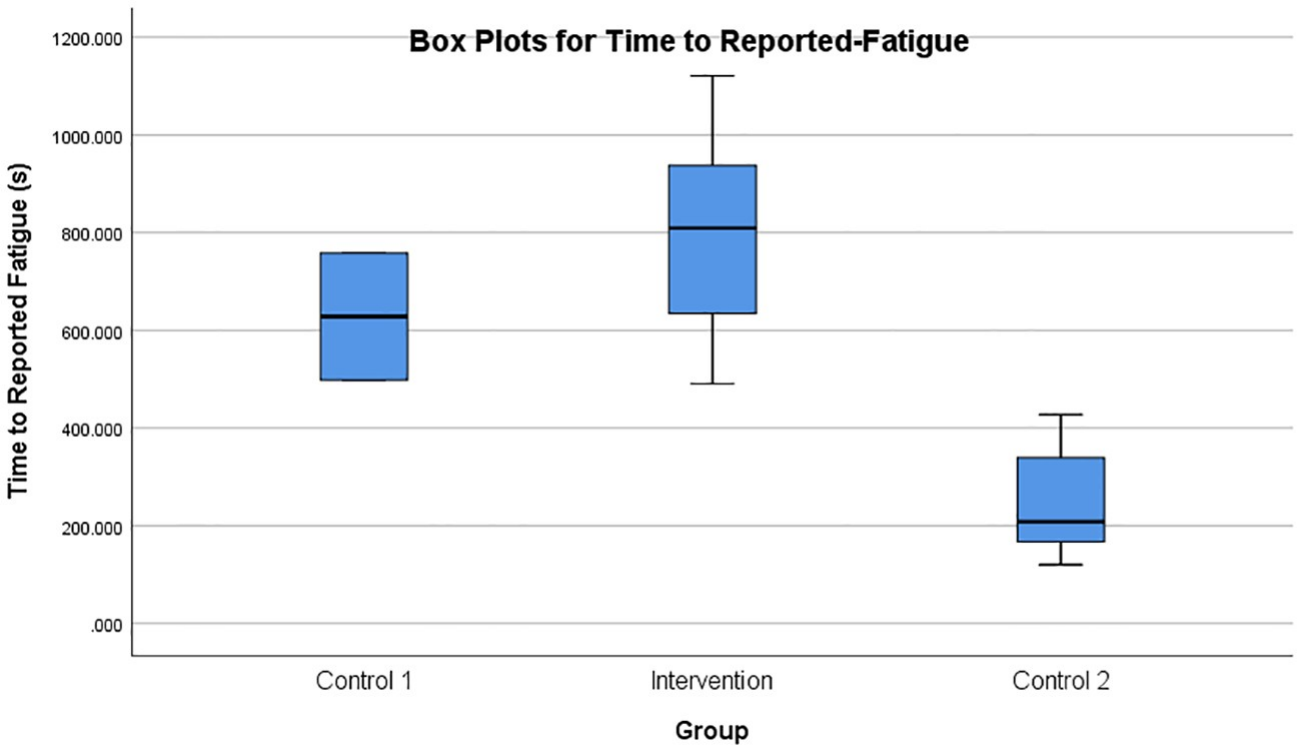
Box plots for time-to-fatigue. Box plots showing the time taken to reach the first reported state of fatigue in the three groups of participants.

The time taken to reach the first reported state of fatigue was also analysed using box plots as shown in Fig 16. The Intervention group was found to have taken more time to reach a state of fatigue, as indicated by the higher values compared to Control 1 and Control 2 groups. This difference was also statistically significant (*F*(2, 12) = 15.778, *p* < 0.005). However, as indicated by *F*(2, 12) in Table 3, not all subjects had a time-to-reported-fatigue value and hence, could not be considered for the ANOVA analysis. Only 2 participants in Control 1 group, 8 participants in the Control 2 group, and 5 participants in the Intervention group reported fatigue after the experiment. Control 1 participants seem to have taken advantage of having the break periods.

During the off-line processing, fatigue flags were also generated using algorithms based on 3-STD, 4-STD and 5-STD thresholds for studying the time of occurrence of the first fatigue. This was then compared against the time of the first reported-fatigue. It was noticed that using a 5-STD threshold for the fatigue-detection algorithm resulted in closer values of time-to-fatigue between the reported and the detected fatigue. As an example, the difference between the time to first-reported-fatigue and the time to first-detected-fatigue considering different thresholds in few subjects was as shown in Table 4. The difference between the two was smaller in the case of the 5-STD threshold compared to the other thresholds in most of the cases. In some cases, the calculated time-gap was negative as few participants reported fatigue before the automatic fatigue detection. However, as seen in Table 4, the time-gap study could not be conducted for few participants. Few subjects did not report any fatigue during the interaction; however, the onset of fatigue could be detected automatically by the algorithm (Intervention and Control 2 groups) and using off-line analysis of EMS (Control 1 group). Also, in few other participants, the EMG median frequencies did not fall below any of the thresholds 3-STD, 4-STD, and 5-STD. Hence, the time-gap values for these subjects could not be shown in the table due to either the absence of any reported-fatigue or detected-fatigue.

**Table 4:** Difference between the the “time to reported-fatigue” and “time to detected-fatigue” in seconds using different thresholds for the fatigue detection algorithm. Time-gap was calculated only for those subjects who had instances of both reported and detected fatigue. For the other participants, the time-gap could not be calculated due to the absence of a reported-fatigue because of the reasons mentioned above. Also, a few other subjects had a negative time-gap due to reporting fatigue prior to automatically detecting fatigue.

Participant No	Group	2-STD	3-STD	4-STD	5-STD
**Subject 1**	Intervention	-248.228	-338.228	-338.228	147.772
**Subject 3**	Control 1	406.949	375.946	375.946	333.946
**Subject 4**	Control 2	-348	NA	NA	NA
**Subject 5**	Control 2	83.545	NA	NA	NA
**Subject 9**	Intervention	841.576	829.574	829.574	829.574
**Subject 12**	Intervention	737.331	707.329	707.329	659.329
**Subject 18**	Intervention	376.438	250.438	16.438	4.438
**Subject 19**	Intervention	268.725	NA	NA	NA
**Subject 21**	Intervention	1043.206	1037.206	1037.206	1013.204
**Subject 22**	Control 2	198.03	198.03	198.03	198.03
**Subject 23**	Control 2	-69.189	NA	NA	NA
**Subject 24**	Control 2	324.039	294.037	288.037	228.037
**Subject 26**	Control 2	277.493	115.493	109.493	79.493
**Subject 30**	Control 2	91.181	49.179	43.179	7.179

#### Correlation results

The correlation statistics between the demographic data and task performance measures for the Intervention group was as shown in Table 5 Corr Intervention. As can be noticed, there was no significant correlation with time-to-fatigue and task repetitions. A moderate correlation was noticed with the task duration. However, for Control 1 (Table 6) and Control 2 (Table 7) groups, only body fat percentage displayed some correlation.

**Table 5:** Correlation statistics for Intervention group. Spearman’s correlation between participant demographic data and the experiment output measurements.

Correlations—Intervention Group						
			Time to Detected Fatigue(s)	Time to Reported Fatigue(s)	Task Rep.	Duration (s)
Spearman’s rho	Skeletal Muscle %	Correlation Coefficient	-0.455	-0.100	0.418	0.673
		Sig. (2-tailed)	0.187	0.873	0.229	0.033
		N	10	5	10	10
	BMI	Correlation Coefficient	0.280	-0.300	-0.170	-0.584
		Sig. (2-tailed)	0.434	0.624	0.638	0.077
		N	10	5	10	10
	Body Fat %	Correlation Coefficient	0.455	-0.300	-0.394	-0.709
		Sig. (2-tailed)	0.187	0.624	0.260	0.022
		N	10	5	10	10

**Table 6:** Correlation statistics for Control 1 group. Spearman’s correlation between participant demographic data and the experiment output measurements.

Correlations—Control 1 Group						
			Time to Detected Fatigue(s)	Time to Reported Fatigue(s)	Task Rep.	Duration (s)
Spearman’s rho	Skeletal Muscle %	Correlation Coefficient	0.700	1.000	0.345	0.50303
		Sig. (2-tailed)	0.188	.	0.328	0.138
		N	5	2	10	10
	BMI	Correlation Coefficient	-0.100	1.000	0.079	-0.515
		Sig. (2-tailed)	0.873	.	0.829	0.128
		N	5	2	10	10
	Body Fat %	Correlation Coefficient	-0.700	-1.000	-0.280	-.766
		Sig. (2-tailed)	0.188	.	0.434	0.010
		N	5	2	10	10

**Table 7:** Correlation statistics for Control 2 group. Spearman’s correlation between participant demographic data and the experiment output measurements.

Correlations—Control 2 Group						
			Time to Detected Fatigue(s)	Time to Reported Fatigue(s)	Task Rep.	Duration (s)
Spearman’s rho	Skeletal Muscle %	Correlation Coefficient	0.600	-0.500	0.503	0.11515
		Sig. (2-tailed)	0.067	0.207	0.138	0.751
		N	10	8	10	10
	BMI	Correlation Coefficient	-0.455	0.048	0.115	-0.248
		Sig. (2-tailed)	0.187	0.911	0.751	0.489
		N	10	8	10	10
	Body Fat %	Correlation Coefficient	-.697	0.262	-0.285	-0.2485
		Sig. (2-tailed)	0.025	0.531	0.425	0.489
		N	10	8	10	10

#### Kinematic data analysis

The kinematic force and position measured by the robot were used to calculate the amount of work done against the resistive forces. The work was calculated using the displacement and the corresponding change in force, as shown in Eq 4. For Control 1 group, the kinematic data during the break periods were removed.

$$\begin{array}{c} Work=\left(F_{x2}-F_{x1}\right)*\left(d_{x2}-d_{x1}\right)+\left(F_{y2}-F_{y1}\right)*\left(d_{y2}-d_{y1}\right)+\left(F_{z2}-F_{z1}\right)*\left(d_{z2}-d_{z1}\right) \end{array}$$ (4)

where F indicates the force applied and d shows the position of the robotic end effector. The notations x, y, and z indicate the three coordinates.

The calculated work was not normally distributed, and also, the study involved more than two groups. Hence, a non-parametric test (Kruskal-Wallis test) was conducted using SPSS to compare the median values across the three subject groups. The resultant Kruskal-Wallis H parameter (approximately chi-square distributed) was used to calculate the effect size estimate using Eq 5. The effect-size indicates the percentage of the variability in the work-done which could be explained by the three groups.

$$\begin{array}{c} EffectSize=\frac{H}{N-1} \end{array}$$ (5)

where H indicates the Kruskal-Wallis H parameter and N indicates the total number of samples.

The non-parametric (Kruskal-Wallis) test results were as shown in Table 8. Test statistic considering all the three groups only gave an effect-size of 5.53%. However, this cannot tell us which specific groups of the independent variable are significantly different from each other; instead, this only tells us that at least two groups were different. To determine how the three groups differ from each other, a specific comparison testing (Kruskal-Wallis test considering two groups each) was also conducted between the groups. As shown in Table 10, the effect-size for the cases “Control 1—Control 2”, “Control 2—Intervention”, and “Control 1—Intervention” were 0.35%, 3.99% and 5.90% respectively. A medians analysis was also conducted to know about the differences in a more easily interpreted way. Comparison of medians of the amount of work by each group of participants was as shown in Table 9.

**Table 8:** Comparing the medians across the three participant groups for the work done against resistive forces using non-parametric (Kruskal-Wallis) test.

**Kruskal-Wallis Test**				**Test Statistics**^a^^,^^b^	
**Ranks**					Work
	Group	N	Mean Rank	Kruskal-Wallis H	9979.187
**Work**	Control 1	39977	105521.04	df	2
	Control 2	51385	99509.26	Asymp. Sig.	0.000
	Intervention	89089	78008.22		
	Total	180451			
**Ranks**				**Test Statistics**^a^^,^^b^	
	Group	N	Mean Rank		Work
**Work**	Control 1	39977	47461.59	Kruskal-Wallis H	323.795
	Control 2	51385	44296.61	df	1
	Total	91362		Asymp. Sig.	0.000
**Ranks**				**Test Statistics**^a^^,^^b^	
	Group	N	Mean Rank		Work
**Work**	Control 2	51385	80905.65	Kruskal-Wallis H	5607.545
	Intervention	89089	64084.29	df	1
	Total	140474		Asymp. Sig.	0.000
**Ranks**				**Test Statistics**^a^^,^^b^	
	Group	N	Mean Rank		Work
**Work**	Control 1	39977	78048.45	Kruskal-Wallis H	7620.456
	Intervention	89089	58468.92	df	1
	Total	129066		Asymp. Sig.	0.000

^a^. Kruskal Wallis Test

^b^. Grouping Variable: Group

**Table 9:** Medians of work done across the three groups of participants. Work is presented here in the units of N.cm.

Comparison of Medians (of Work)—Statistics			
**Control 1**	**N**	Valid	39977
		Missing	72
	**Median**		-0.292193
	**Std. Deviation**		14.3282450
**Control 2**	**N**	Valid	51385
		Missing	1
	**Median**		-0.734028
	**Std. Deviation**		17.1379455
**Intervention**	**N**	Valid	89089
		Missing	2
	**Median**		-3.652203
	**Std. Deviation**		29.8874363

**Table 10:** Effect size estimate calculated using Eq 5.

Effect size estimate	
Control 1, Control 2, Intervention	5.530
Control 1, Control 2	0.354
Control 2, Intervention	3.992
Control 1, Intervention	5.904

## Discussion

The results from Control-2 group showed that the manually reported fatigue in the majority of cases happened a few minutes after the automatically detected fatigue. The algorithm used for the Intervention group for automatic adaptation of the difficulty level was based only on the detected fatigue. Hence, the first detected fatigue did not involve any effects of adaptation. The adaptation would start working only after the first detection of fatigue. Then the difficulty level was reduced and, thus, the participant was assisted in performing the tasks more easily. The intention of having Control-2 group participants was to study if the automatic fatigue detection algorithm in the Intervention group detected the fatigue sooner or later than the reported fatigue. The same algorithm was used in Control-2 group to adjust the difficulty level, but this was based on the reported fatigue; instead of the detected fatigue. In the majority of the cases, the detected fatigue occurred before the reported muscle fatigue during the interaction. This was anticipated because EMG can give a direct measure of the muscle activation, and the muscles may start showing the indication of fatigue onset through the EMG features directly. Hence, the detection of muscle fatigue based on EMG features can be earlier than the actual perception of fatigue. The subjective reporting of muscle fatigue will usually happen when the subject feels pain or is unable to continue after trying his/her level best. Since the reported fatigue happened minutes later than the detected fatigue, the time of occurrence of the reported fatigue was found to have also been influenced by the adaptation process because the reduced difficulty helped the participants to do more iterations.

Control-1 group participants performed the rowing task with difficulty due to the progressively increasing robotic resistance after each 1-minute trial. Some Control-1 group participants even though they performed a difficult task without any assistance from the robot, did not report high fatigue. The subjects were observed to perform the task with a smaller number of repetitions, lesser duration and slower speed of task execution, as shown in Figs 13 and 14. Also, the break period of 30 seconds after each 1-minute trial seemed to have resulted in a recovery of the involved muscles. However, as shown by the progress of damping coefficients in Fig 10, Control-1 group subjects faced a progressively increasing difficulty level after each 1-minute trial.

The Intervention group participants switched between a state of fatigue and relaxation throughout the experiment (Fig 9). Hence, they could perform a large number of iterations with higher speed of execution as shown in Figs 13, 14 and 15. However, most of the participants reported fatigue after the prolonged robotic interaction. But this was at the cost of an increased number of task iterations and an increased duration of the exercise. Even though multiple muscles were involved in the movements some of the muscles were in a prolonged state of fatigue (for example, DLTM muscle in Fig 9), and hence, had a major influence on the final decision on fatigue detection. This could be because the DLTM muscle had a higher involvement in the particular movement in Subject 20, and this could vary across different subjects based on their muscle composition.

In Control-2 group participants, the robotic adaptation happened based on the manually reported fatigue during the task, and the corresponding changes in the task difficulty level were explained by Fig 11. Since the reported fatigue always happened after the automatically detected fatigue, the participants in the Control-2 group always received the task adaptation later than that for the Intervention group participants. Hence, the Control-2 group subjects were finding it more difficult to achieve more iterations and a prolonged interaction, as explained in Figs 13 and 14.

The increased effort in performing the high difficulty task in Control-1 group participants was compensated by reducing the task repetitions and a reduced duration of the experiment. Even though the fatigue rate in the Control-1 group was not as high as expected, the duration for which the participants could perform the progressive strength training task was considerably small. A similar observation was made for the number of task repetitions. However, the amount of work done by the participants seems to be comparable across the groups. The results of non-parametric median comparison (Kruskal-Wallis test) as shown in Tables 8 and 10 showed that there was no much effect of the three groups on the work done against the resistive forces. The effect-size for all the groups were too low, which indicated that only a very low percentage of the variability in the resultant work was explained by the different groups. Comparison of medians of the amount of work also showed that the values were not so different across the groups. This showed that the total work done against the resistive forces across the groups could be compared. Hence, the higher number of task repetitions and the duration in the Intervention group were not due to the lesser amount of work done by these participants. The results also prove that the subjects in the control groups did not give up early because of a superior amount of work done by these participants. As many studies have stated, the number of task repetitions is one of the important criteria for better stroke rehabilitation [12, 13, 59, 60, 61, 9, 25, 11]. Here, it was noticed that the task repetitions could be significantly increased in the Intervention group participants by using a fatigue-adaptive training environment compared to both the manual-adaptive (Control-2 group) and the non-adaptive (Control-1 group) cases.

In general, the results from time-to-fatigue analysis showed that using a 5-STD threshold for fatigue detection resulted in a better match between the time of reported and detected fatigue, as shown in Table 4. However, the time between the reported fatigue and the first detected fatigue (time gap) could not be calculated for all the subjects, since many participants did not manually report fatigue during the sessions. This could be because these participants adapted their effort to the increased task difficulty by reducing the speed and number of task repetitions. This is identified as a limitation and could have been avoided by forcing the subjects to keep a constant pace with an acoustic feedback. However, few among these participants did report fatigue at the end of the tasks through the final questionnaire, even though they did not report this during the robotic interactions. This resulted in not being able to calculate the time-to-reported-fatigue for these participants accordingly, and hence, the time gap could not be calculated. Also, the algorithm needs to be improved to make sure pre-experiment fatigue conditions are considered sufficiently. If a participant is already in a state of fatigue before starting the experiment, the baseline threshold may not be a true representative of the range of EMG feature corresponding to a no-fatigue situation. Therefore, the variation in their median frequency during the task may not be statistically significant to detect muscle fatigue based on the 2-STD-based threshold.

The results from the analysis could not find any particular correlation of the “time-to-fatigue” with the demographic parameters such as age, body weight, Body Mass Index (BMI), visceral fat classification, skeletal muscle percentage or body fat percentage. This seems to be in alignment with the findings of Ma et al. [62], where no significant effects of BMI, muscle mass or age was noticed on the joint muscle strength; instead, this was dependent on the different compositions of muscle fibre types. Ma et al. also stated that fatigue rates were positively correlated with maximum joint moment strength, and higher joint moment strength resulted in faster fatigability in the muscles. The correlation study of the demographic data against time-to-fatigue and experiment duration across the three groups did not show a significant correlation. The intervention group was expected to show the least correlation between the total exercise time and the muscle composition since there would be more frequent switches between fatigued and relaxed states due to the robotic adaptation. On the other hand, the control groups 1 and 2 should show more correlation, since there was no automatic adaptation involved. A person with more physical strength should ideally be able to do more iterations and hence, achieve longer experiment duration. However, the correlation statistics (Tables 5, 6 and 7) for the three subject groups showed that the correlation was not significant in the majority of the cases. For Intervention group, there was a moderate correlation between the demographic data and the task duration. BMI and skeletal muscle percentage had some correlation with task duration only in the Intervention group. For body fat percentage, both Intervention and Control 1 groups showed a negative correlation with the task duration. However, this was not significant in Control 2 group. Overall results indicated that there was no significant influence of demographic data (mainly the skeletal muscle composition and BMI) on time-to-fatigue measurements in the three groups. This could probably be due to the standardisation using MVC-Eq to set the initial task difficulty level.

For Control 1 group, during the break period between different trials, the EMG data acquisition was stopped after each trial. Hence, the different data files (mat files) had to be merged for studying the progress of the EMG features during the off-line processing. This could have been avoided by pausing the data acquisition during the break; instead of stopping the whole data acquisition. It was also noticed that at the end of the experiment, especially for Control-1 group, the iterations were very slow and, hence, the chosen window/buffer size corresponding to 6 seconds does not seem to include the EMG corresponding to a full rowing task completely. This resulted in the calculated EMG features at the end stage of the experiment, not representing the muscle activations corresponding to a complete rowing movement. The features might have a different composition compared to that of a full cycle of rowing task. This was due to the fixed buffer size of 6 seconds.

It was noticed that many subjects in the Intervention group reported “relaxed” not too late after receiving the robotic adaptation. It could be that the fatigue detected by the current algorithm based on the 2-STD threshold might be an indicator of just the onset of fatigue and not that of a high state of fatigue. Hence, the time taken to come to a state of relaxation was not too long. There are various applications for the automatic fatigue detection algorithm like in human-robot interaction, rehabilitation training, muscle building or strength training exercises. Here, the detected muscle fatigue may be utilised in different ways, for example, for delaying/avoiding fatigue or causing fatigue. As explained in Table 4, depending on the application, the fatigue threshold may be adjusted such that the detected fatigue comes closer to the reported fatigue. Increasing the fatigue threshold to higher values like 5-STD threshold could be more accurate to be used in algorithms that adapt based on a high level of fatigue. However, more explorations need to be done in this area. Some recent studies have also reported that despite a reasonable rest period after a fatiguing protocol, while the performance recovered, there was even further progression of fatigue when measured by EMG. Hence, it would also be interesting to look at different time-frames following the fatigue protocol to underpin the time required for sufficient recovery from fatigue during a robot-assisted interaction.

In the current study, the proposed method was tested with healthy participants, and hence, the suitability of using this method for actual rehabilitation need to be explored further. Before introducing this approach to the stroke group in future, we intend to adjust further and evaluate our methods using a longitudinal study that targets and observes the various stages of stroke recovery. The process of motor recovery after stroke can be broken down into seven stages as per Brunnstrom’s approach [63, 64]. During Stage 1, a stroke survivor cannot initiate any muscle movements on the affected side of their body. If this continues for a long time without intervention or physical therapy, the unused muscles can become much weaker. Stage 1 condition also requires lifestyle modifications to protect the affected limbs from injury. In Stage 2, redevelopment of some basic muscle synergies occurs. Muscles begin to make small, spastic, and abnormal movements during this stage. These movements are mostly involuntary, but they can be a promising sign during the recovery. Many stroke survivors also experience spasticity at this stage, which presents resistance to passive movement, and results in jerky movements. It is important to continue using and moving the muscles as much as possible at this stage. Spasticity of muscles increases in Stage 3, feeling unusually stiff, tight, or pulled muscles. This causes an abnormal increase in muscle stiffness. An increase in involuntary movements occur due to the ability to initiate a muscle movement, but not able to control it yet. Hence, the patients in Stage 3 are limited in their ability to exercise and may require help to do this. So, it is important to minimise highly stressful activities at this stage. Passive range of motion exercises shall be continued at this stage to improve the range of motion. The main focus during Stage 4 is to strengthen and improve muscle control. Spastic muscle movements start declining at this stage, and the patient begins making normal or controlled movements on a limited basis. Active-assisted range of motion exercises are used at this stage since the patients get some ability to move but still needs help to practise the exercises or complete the movement. Afterwards, active range-of-motion exercises can also be started once the patient gains some muscle control. They can then perform some exercises without assistance. In stage 5, spasticity continues to decline, and muscle synergy patterns become more coordinated, allowing controlled and deliberate voluntary movements. These purposeful and goal-directed movements can improve with repetition and practice. In Stage 6, muscle spasticity disappears completely, the patient will be able to move individual joints, and the synergy patterns become much more coordinated. Motor control will be almost fully restored, allowing the patient to coordinate complex reaching movements. Finally, during Stage 7, the patient regains the full motor function in the affected areas [63, 64]. The process of stroke rehabilitation as it goes through these different stages has to deal with acute adaptation (e.g. fatigue induced by the exercise) as well as chronic adaptations (e.g. increased strength over time) to training. So, while extending this protocol for stroke rehabilitation, the suggested training protocol should be tuned to these different stages of the recovery process.

The patterns of acute as well as chronic adaptations to training could be an interesting future work along this line. Resistance training adaptations can entail both acute and chronic adaptation. The acute physiological response causes an immediate change (increase or decrease) in the body systems in response to a stimulus. Acute responses to resistance training occur primarily in the neurological, muscular, and endocrine systems [65]. Acute neurological adaptations during resistance training can cause the muscles to grow more tired with each repetition of a given movement pattern. As a result, the motor unit firing sequence becomes less and less precise. One of the acute muscular adaptations is the depletion of metabolic substrates, which causes a decrease in muscle power production. Also, an increase in the intramuscular hydrogen causes a “burning” sensation in the muscles on multiple repetitions [66]. On the other hand, chronic physiological adaptations cause changes to the body systems as a result of long term stimulus, such as exercise. Chronic adaptations can occur if the exercise is repeated on several occasions, and this depends on the type of exercise. This can make the muscle more resistant to fatigue, stronger, more powerful, or better coordinated. Chronic adaptations involve either remodelling of tissue or altered regulation of the central nervous system [65]. Chronic responses to resistance training are seen in the muscular, skeletal, cardiovascular, neurological systems, and body composition. Chronic neurological adaptations result in a more efficient sequence of recruitment of motor units, making the muscle less apt to tire from neuromuscular factors. Another chronic adaptation to the neurological system is an increased motor unit firing and decreased co-contraction of the antagonistic muscles. This allows for greater movement efficiency. Chronic muscular adaptations to resistance training include the increased cross-sectional size of the muscle fibres, which increases muscle strength and power. Also, adaptations of the skeletal system occur over the long term, and changes in body composition are also seen as a chronic adaptation to resistance training [66].

The results from the current study showed that muscle engagement in the exercise could be regulated by the adaptive action of a robot, and fatigue-adaptive robotic systems can potentially help to prolong training interactions. However, as a pilot study, this manuscript has only addressed acute adaptations during the interaction. Hence, more work is needed (i.e. a longitudinal/training study) to prove that the method can be used in a rehabilitation program, by considering chronic adaptations over a long duration of robot-assisted training.

## Conclusions

Progressive strength training has been suggested in stroke rehabilitation protocols by many past studies. In this study, an adaptive robotic interaction protocol for muscle training was suggested to be used in rehabilitation training. The robotic assistance could be used for an adaptive interaction by switching between different intensities based on the detected muscle fatigue. The results indicated that a progressive increase of task difficulty and adaptation of the difficulty level based on the onset of muscle fatigue resulted in a prolonged training interaction and increased task repetitions. The features derived from the EMG measurements from the upper limb muscles were found effective to be used as fatigue indicators for adaptive rehabilitation training.
